# Identification of the bacterial metabolite aerugine as potential trigger of human dopaminergic neurodegeneration

**DOI:** 10.1016/j.envint.2023.108229

**Published:** 2023-09-23

**Authors:** Anna-Katharina Ückert, Sina Rütschlin, Simon Gutbier, Nathalie Christine Wörz, Mahfuzur R. Miah, Airton C. Martins, Isa Hauer, Anna-Katharina Holzer, Birthe Meyburg, Ann-Kathrin Mix, Christof Hauck, Michael Aschner, Thomas Böttcher, Marcel Leist

**Affiliations:** a In vitro Toxicology and Biomedicine, Dept inaugurated by the Doerenkamp-Zbinden foundation, University of Konstanz, 78457 Konstanz, Germany; b Department of Chemistry, Konstanz Research School Chemical Biology, Zukunftskolleg, University of Konstanz, 78457 Konstanz, Germany; c Faculty of Chemistry, Institute for Biological Chemistry & Centre for Microbiology and Environmental Systems Science, Department of Microbiology and Ecosystems Science, University of Vienna, Josef-Holaubek-Platz 2 (UZA II), 1090 Vienna, Austria; d Doctoral School in Chemistry (DoSChem), University of Vienna, 1090 Vienna, Austria; e Department of Molecular Pharmacology, Albert Einstein College of Medicine, 10641 Bronx, NY, United States; f Department of Neuroscience, Albert Einstein College of Medicine, 10641 Bronx, NY, United States; g Lehrstuhl Zellbiologie, Universität Konstanz, Universitätsstraße 10, Postablage 621, 78457 Konstanz, Germany

**Keywords:** Dopaminergic neurodegeneration, *Streptomyces venezuelae*, Bacterial toxin, Ferroptosis, *Caenorhabditis elegans*, Parkinson’s disease

## Abstract

The causes of nigrostriatal cell death in idiopathic Parkinson’s disease are unknown, but exposure to toxic chemicals may play some role. We followed up here on suggestions that bacterial secondary metabolites might be selectively cytotoxic to dopaminergic neurons. Extracts from *Streptomyces venezuelae* were found to kill human dopaminergic neurons (LUHMES cells). Utilizing this model system as a bioassay, we identified a bacterial metabolite known as aerugine (C_10_H_11_NO_2_S; 2-[4-(hydroxymethyl)-4,5-dihydro-1,3-thiazol-2-yl]phenol) and confirmed this finding by chemical re-synthesis. This 2-hydroxyphenyl-thiazoline compound was previously shown to be a product of a wide-spread biosynthetic cluster also found in the human microbiome and in several pathogens. Aerugine triggered half-maximal dopaminergic neurotoxicity at 3–4 μM. It was less toxic for other neurons (10–20 μM), and non-toxic (at <100 μM) for common human cell lines. Neurotoxicity was completely prevented by several iron chelators, by distinct anti-oxidants and by a caspase inhibitor. In the *Caenorhabditis elegans* model organism, general survival was not affected by aerugine concentrations up to 100 μM. When transgenic worms, expressing green fluorescent protein only in their dopamine neurons, were exposed to aerugine, specific neurodegeneration was observed. The toxicant also exerted functional dopaminergic toxicity in nematodes as determined by the “basal slowing response” assay. Thus, our research has unveiled a bacterial metabolite with a remarkably selective toxicity toward human dopaminergic neurons *in vitro* and for the dopaminergic nervous system of *Caenorhabditis elegans in vivo*. These findings suggest that microbe-derived environmental chemicals should be further investigated for their role in the pathogenesis of Parkinson’s disease.

## 1. Introduction

The increasing number of idiopathic Parkinson’s disease (PD) cases suggests that environmental factors exist that specifically affect dopaminergic (DAergic) neuron viability. Indeed, several industrial chemicals and pesticides have been implicated to contribute to PD pathogenesis (Bellou et al., 2016; Obeso et al., 2017; Tanner, 1992; Terron et al., 2018). Metabolites synthesized by bacteria and fungi are also candidates for initiation and perpetuation of the disease (Dorsey et al., 2018; Fuhrimann et al., 2021; Inamdar et al., 2013).

Although some genes are known to be associated with PD as dominant or recessive factors, at least 90% of all PD cases are of sporadic origin (Obeso et al., 2017; Program, 2021). Several lines of evidence, including twin studies, have shown the critical importance of environmental factors regarding PD onset. Age is clearly the major risk factor for PD, but it is not clear, why part of the aging population develops the disease and a major fraction does not (Costello et al., 2009; De Miranda et al., 2022; Goldman, 2014; Priyadarshi et al., 2001; Yuan et al., 2022).

There is a wealth of epidemiological data connecting pesticide exposure to PD (Kanthasamy et al., 2012). The case is particularly convincing for the pesticide rotenone, and this is well supported by mechanistic studies and animal experiments (Betarbet et al., 2000; De Miranda et al., 2022; Ockleford et al., 2017). Some evidence suggests also a role for dieldrin (Hatcher et al., 2007; Moretto and Colosio, 2011) and high levels of paraquat (McCormack et al., 2005; Ockleford et al., 2017), but the final verdict on these is still pending. Whilst pesticides might be responsible for some of the PD cases in rural areas, overall disease prevalence is unlikely to be explained by exposure to agricultural chemicals (Costello et al., 2009; Firestone et al., 2005; Gorell et al., 1998; Priyadarshi et al., 2001). The same applies to manganese exposure (Austin et al., 2016; Erikson and Aschner, 2019). Another chemical capable of inducing parkinsonism is 1-methyl-4-phenyl-1,2,3,6-tetrahydropyridin (MPTP), but only few cases of drug addicts have been documented (Langston, 2017).

Based on our knowledge on disease etiology, other chemicals may play roles in PD etiology by promoting neuroinflammation or ferroptosis (de Farias et al., 2017; Panicker et al., 2022; Sian-Hulsmann and Riederer, 2021). Neuroinflammatory conditions in the developing brain have been shown to contribute to the loss of dopamine (DA) neurons (Barlow et al., 2007). Similarly, in the adult brain, the activation of glial cells and the presence of certain cytokines have been identified as potent exacerbators of nigrostriatal degeneration in animal models (Schildknecht et al., 2017). However, environmental chemicals triggering such conditions remain elusive. The situation is similar for changes in the homeostasis of free ferric and ferrous ions in cells. Iron can play an important role in DAergic cell death (Devos et al., 2014; Mahoney-Sánchez et al., 2021), but the specific chemical triggers for such changes remain unknown.

Metabolites produced by the microbiome have been considered relevant factors, as they may affect large populations. Indeed, the gut microbiome of PD patients is different from healthy individuals, and the gut microbiome-brain axis has been shown to be altered in PD (De Miranda et al., 2022). Moreover, neurodegenerative changes of the enteric nervous system occur during early stages of PD pathology. They may predate the onset of motor symptoms by years (Braak et al., 2006; Scheperjans et al., 2015; Shannon et al., 2012). In experimental animals, a link between gut microbiota and PD pathology has been established, as gut stool transferred from PD patients promoted motor dysfunction in mice (Sampson et al., 2016).

Some microbial metabolites have been reported to specifically affect DAergic neurons. For instance, the fungal metabolite 1-octen-3-ol was active in a *Drosophila melanogaster*-based PD model (Inamdar et al., 2013). Moreover, *Streptomyces venezuelae* was suspected to produce a metabolite, which caused selective DAergic neurodegeneration in *Caenorhabditis elegans* (Caldwell et al., 2009). The bioactivity of the putative metabolite was characterized extensively, but the exact molecular identity has not been elucidated (Caldwell et al., 2018; Martinez et al., 2015; Ray et al., 2014).

The toxin production of *S. venezuelae* may be of limited relevance for most PD cases, as these bacteria typically live in soil. However, elucidation of the neurotoxicant structure can help in identifying the genes involved in its synthesis. Biosynthetic clusters for secondary metabolites often use simple primary metabolites (*e.g.,* amino acids) to build complex new molecules (Inahashi et al., 2017; Vior et al., 2018), and the genes for this process can be transmitted horizontally across many bacterial species (Tidjani et al., 2019). Thus, secondary metabolites identified in soil bacteria may also be found in microbes closely associated with human physiology or pathology (Abe et al., 2020; Jiang et al., 2017).

An important condition for the identification of DAergic toxicants are suitable model systems. The use of *C. elegans* is well established for studies of the molecular pathology of PD (Cooper et al., 2006; Liu et al., 2011; Tucci et al., 2011). The model has also been used successfully in neurotoxicity testing (Braungart et al., 2004; Caito et al., 2013; Vanduyn et al., 2010). A complementary approach makes use of DAergic neurons generated from induced pluripotent stem cells (iPSC), or of LUHMES cells, *i.e.* conditionally immortalized human DAergic precursor cells that can be triggered to undergo terminal differentiation to post-mitotic neurons (Scholz et al., 2011). The latter cells are highly sensitive to various neurotoxicants, and have been characterized to undergo apoptosis, ferroptosis or selective neurite degeneration, depending on the toxicant used (Delp et al., 2019; Efremova et al., 2015; Gutbier et al., 2018a; Gutbier et al., 2018b).

Being intrigued by a potentially specific DAergic toxicant produced by microbes (Caldwell et al., 2018; Caldwell et al., 2009), we set out to verify this hypothesis and to elucidate the chemical structure. Our initial studies confirmed that *S. venezuelae* extracts killed human, DAergic neurons, while the related strain *S. lividans* did not have such an effect. A purification strategy based on a cellular neurotoxicity assay was developed. After identification of the bioactive molecule, it was re-synthesized and tested for selective neurotoxicity to human neurons. A pharmacological rescue screen was undertaken to profile the type of cell death triggered. Finally, we aimed to determine toxicant specificity for DAergic neurons *in vivo* by using the *C. elegans* model system.

## 2. Material and methods

### 2.1. Materials and chemicals

Dibutyryl cyclic adenosine monophosphate (dBcAMP), fibronectin, fetal calf serum (FCS), Hoechst H-33342, poly-L-ornithine hydrobromide (PLO), resazurin sodium salt, tetracycline, sodium pyruvate, and Triton-X100 were purchased from Sigma Aldrich (Steinheim, Germany). Chemicals and solvents used for extraction, purification and synthesis of aeruginol and derivatives were purchased from Sigma Aldrich (Steinheim, Germany). Nicotinamide-adenine-dinucleotide (NADH) disodium salt was purchase from Carl Roth GmbH + Co. KG (Karlsruhe, Germany). Recombinant human fibroblast growth factor 2 (FGF-2) and recombinant human glial cell derived neurotrophic factor (GDNF) were purchased from R&D Systems (Minneapolis). All cell culture reagents were purchased from Gibco/Fisher scientific (Hampton, New Hampshire, USA) unless otherwise specified. Seahorse materials and reagents were purchased from Agilent (California, United States). Cortex.4U, CNS.4U and their culture media were purchased from Axiogenesis (Köln, Germany). Dihydroaeruginoic acid (DHAA, CAS 143209–04-5) was purchased from Santa Cruz Biotechnology, Inc (Dallas, US). Calcein-AM was purchased from Biomol GmbH (Hamburg, Germany).

### 2.2. Extraction and purification of Streptomyces metabolites

*S. venezuelae* (ATCC 10712) and *S. lividans* (ATCC 19844) were grown in SYZ medium (15 g/l starch, 2 g/l yeast extract, 4 g/l casein peptone, 2 g/l glucose, pH = 6.2) at 30 °C and 160 rpm over 3 days and 350 μl were inoculated on a large (145 × 20 mm) SYZ agar plate (SYZ medium + 15 g/l agar). The plate was incubated for 4 days at 30 °C. The bacteria were scratched off with a spatula from the surface and resuspended in 5 ml SYZ medium. 200 μl of the bacterial suspension were then added onto 40 large (145 × 20 mm) SYZ agar plates and the plates incubated for 6 days at 30 °C. The plates were chopped into pieces and ethyl acetate (EA) was added. The extraction was repeated 3 times with 2 l of EA each and the solvent was evaporated. SepPak fractionation (5 g cartridge, Sep-Pak C18 20 cc, 55–105 μm particle size, Waters) and elution with 0, 30, 60 and 100% methanol (MeOH) yielded an active 60% MeOH fraction. The active fraction was further sub-fractionated by preparative reverse phase HPLC on a C18-AQ column with Reprosil-Pur 120 (250 × 20 mm, 10 μm, Dr. Maisch) using the following gradient: T_0min_ = 50% B, T_3min_ = 50% B, T_25min_ = 95% B, with a flow rate of 15 ml/ min, monitoring absorbance at 195 nm. The solvents were: A = water (+0.1% formic acid (FA)) and B = acetonitrile (+0.1% FA). At 17 min, an active fraction eluted which was further purified *via* semi-preparative reverse phase HPLC on a C18 column with Reprospher 100 (250 × 10 mm, 5 μm, Dr. Maisch) using a flow rate of 1.5 ml/min, monitoring absorbance at 254 nm. Gradient: T_0min_ = 70% B, T_1min_ = 70% B, T_40 min_ = 95% B. The solvents were: A = water (+0.1% FA) and B = acetonitrile (+0.1% FA). NMR measurements indicated a mixture of two compounds, which were separate again by semi-preparative HPLC using the same column. Gradient: T_0min_ = 30% B, T_5min_ = 30% B, T_25min_ = 80% B, T_40 min_ =95% B. After NMR and MS characterization, aerugine (0.68 mg) and aeruginol (0.46 mg) could be unambiguously confirmed by NMR spectroscopy and mass spectrometry and comparison to literature values.

### 2.3. LUHMES cell culture

LUHMES cells were cultivated as described previously (Krug et al., 2013; Schildknecht et al., 2013; Scholz et al., 2011). In brief, cells were grown in standard cell culture flasks pre-coated with 50 μg/ml PLO and 1 μg/ml fibronectin in water for at least 3 h at 37 °C. The maintenance culture was kept in proliferation medium consisting of advanced DMEM/F12 with 2 mM L-glutamine, 1x N2-supplement, and 40 ng/ml FGF-2. The cells were incubated at 37 °C with 5% CO_2_ and passaged trice a week when reaching 75–90% confluence. They were used up to passage 18. For differentiation, the medium was changed to differentiation medium consisting of advanced DMEM/F12 supplemented with 2 mM L-glutamine, 1 mM dBcAMP, 1 μg/ml tetracycline and 2 ng/ml GDNF on day of differentiation 0 (d0).

For toxicity assessment of mature LUHMES neurons, precursor cells were pre-differentiated for 2 days in cell culture flasks pre-coated as described above and differentiation medium. Then, 45′000 cells per well were seeded in differentiation medium into standard 96-well plates pre-coated as described above. They were incubated at 37 °C and 5% CO_2_ for 4 days before compound treatment. At this time, cells were >99% post-mitotic and had established an extensive neurite network (Scholz et al., 2011) The cell viability was assessed *via* the Calcein/Hoechst readout, the LDH assay and the resazurin reduction assay.

### 2.4. Stem cell derived neurons

The human iPSC (Sigma iPSC0028 (EPITHELIAL-1, #IPSC0028) were maintained and differentiated towards immature dorsal root ganglia neurons according to Hoelting et al. (2016) (Hoelting et al., 2016). Briefly, a total of 90,000 cells/cm^2^ were seeded on Matrigel. The differentiation was initiated with neural differentiation medium (KSR-S; Dulbecco’s modified Eagle’s medium [DMEM/F12] containing 15% knockout serum replacement, 1 × Glutamax, 1 × nonessential amino acids, and 50 μM ß-mercaptoethanol) supplemented with 17.5 ng/ml noggin, 10 μM SB-431642 from day of stem cell differentiation (DoD) DoD0 to DoD5. Starting on DoD2 CHIR99021 (1.5 μM), SU5402 (5 μM) and DAPT (5 μM) were added. From DoD4 onwards, the medium was step-wise replaced by N2-S medium (DMEM/F12 containing 2 mM Glutamax, 0.1 mg/ml apotransferrin, 1.55 mg/ml glucose, 25 mg/ml insulin, 100 mM putrescine, 30 nM selenium, and 20 nM progesterone; 25% N2-S medium on DoD4, 50% N2-S medium on DoD5, 75% N2-S medium on DoD7). On DoD9, the cells were frozen.

Commercial preparation of iPSC-derived neurons (Cortex.4U and CNS.4U) were handled and used as described by the manufacturer (nCardia). In brief, plates were coated with 10 μg/ml PLO, 10 μg/ml laminin and 10 μg/ml fibronectin. Cells were thawed and immediately seeded onto 96-well plates in their respective culture media (Cortex.4U: Cortex.4U^™^ culture medium with Cortex.4U^™^ supplement; CNS.4U: Neuro.4U Basal Medium [Ax-M-NBM250] with Neuro-Supplement 2 [Ax-M-DCS-DA]) to be used in neurite outgrowth assays.

### 2.5. Neurite outgrowth assays

The assays were performed as described previously (Hoelting et al., 2016; Klima et al., 2021; Krug et al., 2013). In brief, 30′000 cells per well were seeded on d2 (LUHMES) / d0 (peripheral neurons, Cortex.4U, CNS.4U) in their respective differentiation media into standard pre-coated 96-well plates. They were incubated for 1 h at 37 °C and 5% CO_2_ before treatment. 24 h after treatment the Calcein/Hoechst readout was performed.

### 2.6. Hek293FT, HepG2 and HeLa cell culture

Hek293FT were acquired from ThermoFisher Scientific (cat. no. R70007). HepG2 were acquired from ATCC (HB-8065). HeLa were acquired from ATCC (CCL-2). The cell culture was performed as described previously (Dolde et al., 2021). Briefly, the cell lines were kept in DMEM Glutamax with 10% FCS and 1% penicillin/streptomycin at 37 °C with 5% CO_2_. They were grown in normal cell culture flasks (Sarstedt) to 75–90% confluence and seeded into standard 96-well plates 24 h prior to compound treatment. The cells were treated by performing a medium change to Advanced DMEM/F12 supplemented with 2 mM L-glutamine containing the indicated compound concentrations.

### 2.7. Calcein/Hoechst readout

Neurite area (NA) and viability (V) were assessed as described previously (Stiegler et al., 2011). In brief, cells were stained with 1 μM calcein-AM and 1 μg/ml H-33342 for 30 min at 37 °C and 5% CO_2_. Image acquisition was performed using an Array-Scan VTI HCS Reader (Cellomics, PA, USA) equipped with a Hamamatsu ORCA-ER camera. 10 fields per well were imaged with 2 channels at 20x magnification (2×2 pixel binning). Excitation/emission wavelengths of 365 ± 50/535 ± 45 nm were used for Hoechst detection in channel 1 and 474 ± 40/535 ± 45 nm were used for calcein detection in channel 2.

Nuclei were identified in channel 1 depending on intensity, area, size and shape. Their outlines were expanded by 3.2 μm to define a virtual cell soma area (VCSA), which was bigger than the average cell soma to prevent false positive NAs. All calcein-positive pixels were defined as viable cellular structures (VCSs). The NA was automatically calculated by excluding the VCSAs from the VCSs. Furthermore all nuclei co-localizing with VCSs were defined as alive.

### 2.8. Additional TMRE/PI readout

Cells were stained either with 50 nM tetramethylrhodamine ethyl ester (TMRE) or 1.5 μM propidium iodide (PI) for at least 30 min at 37 °C and 5% CO_2_ (Brüll et al., 2020; Krug et al., 2014). Fluorescent imaging was performed (AxioObserver, Zeiss, Germany) with excitation/emission wavelengths of 575 ± 25/640 ± 35 nm.

### 2.9. LDH assay

The assay was performed as described earlier (Latta et al., 2000). In brief, supernatants were transferred to an un-coated 96-wellplate and cells were lysed in 110 μl of phosphate buffered saline (PBS) with 0.1% Triton-X100 for 1 h at room temperature. Lactate dehydrogenase (LDH) activity was assessed in the supernatant and the cell lysate individually by combining 10 μl sample with 100 μl potassium phosphate buffer pH 7.5 containing 640 μM pyruvate and 240 mM NADH. NADH was quantified by measuring the absorption at 340 nm over 15 min and its decline was proportional to the LDH content. LDH release was calculated as the ratio of LDH_supernatant_/LDH_supernatant+lysate_.

### 2.10. Resazurin reduction assay

The assay was performed as described previously (Schildknecht et al., 2009). In brief, cells were stained with 10 μg/ml resazurin and incubated for 30 min at 37 °C and 5% CO_2_. The fluorescence of resazurin was detected with an excitation wavelength of 530 nm, using a 590 nm long-pass filter to record the emission.

### 2.11. Seahorse assessment of mitochondrial respiratory parameters

The method was performed as described previously. (Delp et al., 2018) In brief, LUHMES cells were seeded on d2 into pre-coated Agilent Seahorse XFe24 well plates at a density of 100′000 cells/well in differentiation medium. On d3 the cells were analyzed in a Seahorse XF analyzer using the Mito stress test kit in accordance with the manufacturer’s instructions. In brief, 1 h prior to analysis the medium was changed to Seahorse XF base medium supplemented with 18 mM glucose, 2 mM glutamine and 1 mM pyruvate. The oxygen consumption rate (OCR) was measured to determine the basal respiration in untreated cells. After test compound injection the acute response was quantified. Injection of 1 μM oligomycin allowed for the determination of ATP production. 1.5 μM carbonyl cyanide-4-(trifluoromethoxy) phenylhydrazone (FCCP) uncoupled the electron respiratory chain from the ATP synthase and enabled the measurement of the maximal respiration. The addition of 0.5 μM rotenone and 0.5 μM antimycin A completely shut down mitochondrial respiration to quantify non-mitochondrial respiration. Afterwards the cells were stained with 1 μg/ml Hoechst for 30 min at 37 °C. Image acquisition was performed using an Array-Scan VTI HCS Reader (Cellomics, PA, USA) equipped with a Hamamatsu ORCA-ER camera. 25 fields per well were imaged at 20x magnification (2×2 pixel binning). Excitation/emission wavelengths of 365 ± 50/535 ± 45 nm were used for H-33342 detection. Nuclei were identified and quantified depending on intensity, area, size and shape. According to pixel size and well size the total cell count was calculated and used for normalization of the OCR data.

### 2.12. Bacterial growth assay

*Neisseria gonorrhoeae* MS11 was cultured in PPM medium (15 g/l Proteose Pepton, 1 g/l soluble starch, 5 g/l NaCl, 4 g/l KH_2_PO_4,_ 1 g/l K_2_HPO_4,_ pH 7.5) for liquid culture and on GC plates (BD Difco^™^ GC Medium Base), both supplemented with 1 % vitamin mix (100 g/l glucose, 10 g/l glutamine, 26 g/l L-cysteine, 100 mg/l carboxylase, 250 mg/l NAD, 500 μl/l Fe(NO_3_)_3_, 150 mg/l arginine, 3 mg/l thiamine-HCl, 10 mg/l vitamine B12, 13 mg/l p-amino benzoic acid, 1.1 g/l L-cystine, 1 g/l adenine, 500 mg/l uracil, 30 mg/l guanine). Strains were either cultivated at 37 °C and 220 rpm (liquid medium) or at 37 °C and 5 % CO_2_ (solid medium).

*Enterococcus faecalis* was grown in BHI medium (BD Difco Brain Heart Infusion) with 36 g/l supplemented with 20 μg/ml hemin, 20 μg/ml NAD and 15 g/l agar for plates. *Enterococcus faecalis* strain 438 originates from eye isolate. *Neisseria gonorrhoeae* strain MS11 was gifted by T. F. Meyer (Max-Planck-Institute for Infection Biology, Berlin, Germany) (Edwards et al., 1984).

Bacteria were grown as described above on solid medium. Prior to substance exposure, bacteria were pre-cultured in liquid medium for at least 2 h. Optical density was determined and a volume equal to an OD550 of 0.2 for *Neisseria gonorrhoeae* MS11 (equates a final OD550 of 0.04) and an OD600 of 0.2 for *Enterococcus faecalis* (equates a final OD600 of 0.04) was inoculated into 5 ml of the respective liquid medium. Compound was added at the indicated amounts. Dimethyl sulfoxide (DMSO) level was adjusted to 1 % of final concentration including DMSO control. Samples were incubated for either 10.5 h, until they reached a stationary phase (three measured values in stationary phase) or until they reached an OD of 2.5. Optical density was determined every 0.5 h at OD550 for *Neisseria gonorrhoeae* MS11 and at OD600 for *Enterococcus faecalis*. (Szamosvári et al., 2019).

### 2.13. C. elegans study to determine specific neurodegeneration and behavioral changes

The strain BY200 Pdat-1::gfp (vtIs1), dat-1::GFP(vtls1) was kindly provided by the Blakely laboratory, Vanderbilt University Medical Center and the strain Punc-25:GFP was obtained from the Caenorhabditis Genetics Center (CGC, University of Minnesota, Minneapolis, MN, USA). The worms were maintained in nematode growth medium (NGM) plates that contain: 3 g/l NaCl, 17 g/l agar, 2.5 g/l peptone, 1 mM CaCl_2_, 5 mg/l cholesterol, 1 mM MgSO_4_, 25 mM potassium phosphate buffer (pH 6.0) with 1.25 ml nystatin and 50 mg/l streptomycin sulfate) and *Escherichia coli* (*E*. *coli*) OP50 strain was used as food (Brenner, 1974). Synchronization of worms was carried out using extraction solution (0.2% NaOCl, 0.5 M NaOH, in distilled water). The first larval stage (L1) population were obtained by isolating eggs with a 30% sucrose gradient, washed with sterile water, and resuspended in M9 buffer (42 mM Na_2_HPO_4_, 22 mM KH_2_PO_4_, 8.5 mM NaCl, and 1 mM MgSO_4_) (Sulston and Hodgkin, 1998).

For the lethality analysis, 40 to 60 BY200 and P unc-25 worms at L4 larval stage were exposed to vehicle or aerugine (8, 40 and 100 μM) in OP50-seeded NGM plates from day 0 until day 8. Worms were scored as dead when they did not respond to a mechanical stimulus. Moreover, worms were transferred to fresh plates for the first three days of treatment, which coincide with the egg laying days to avoid mixing of different generations. All treatments were performed in triplicates and experiments were repeated independently at least three times.

The basal slowing response (BSR) is a behavioral assay that assesses the functionality of the worms’ DAergic system. For this, worms were washed three times with S-basal buffer (100 mM NaCl, 5 mg/L cholesterol, 50 mM potassium phosphate buffer, pH 6.0) to remove residual bacteria, and placed on the center of a 60-mm NGM plates in the absence or presence of an OP50 bacteria ring. After habituation (5 min), the number of body bends of each worm was counted during 20 s to determine its immobility rate. The results were expressed as the difference (Δ) between the number of body bends in the absence (a) and presence (p) of bacteria on fresh plates (Δ = a-p). Given, worms with normal DA content move slower in the presence of bacteria than in the absence of bacteria (Sawin et al., 2000).

On day 2 and day 8, approximately, 20–30 worms per treatment were picked onto slides prepped with a 3% agarose pad, anesthetized with 5 μl of 3 mM levamisole hydrochloride (Sulston et al., 1975) and observed under an Olympus BX41 fluorescence microscope to determine morphology status according to novel Likert-like scale of degeneration as previously described (Benedetto et al., 2010).

### 2.14. Chemical analysis and identification of aerugine and aeruginol

^1^H NMR and ^13^C NMR spectra were either recorded on a Bruker Avance III 400 (400 MHz) or a Bruker Avance III 600 (600 MHz) spectrometer and referenced to the residual proton and carbon signal of the deuterated solvent, respectively.

LC-MS analysis was conducted with a Bruker Amazon SL IonTrap mass spectrometer connected to a 1260 Infinity Agilent Technologies HPLC equipped with a Nucleoshell RP-18 column (50 × 2 mm, 2.7 μm) (Macherey-Nagel, Germany) operating in positive ion mode with a flow rate of 0.5 ml/min. For 30 s the flow was disconnected from the ESI source and flushed into the waste to prevent salts from entering the MS. Mobile phases for the mass spectrometers was A = water (+0.1% FA) and B = acetonitrile (+0.1% FA). The gradient was: T_0 min_: B = 5%; T_5 min_: B = 5%; T_25 min_: B = 95%; T_33 min_: B = 95%.

Aerugine


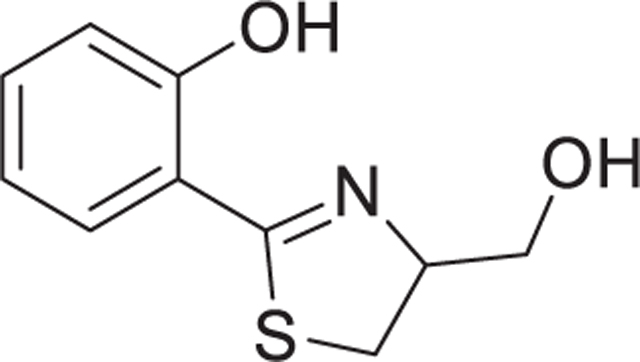


^1^H NMR (800 MHz, DMSO-*d*_6_) *δ* 12.65 (s, 1H, Ph-O**H**), 7.41 (t, *J* = 7.3 Hz, 2H, Ph**-H**), 6.97 (dd, *J* = 8.9, 1.2 Hz, 1H, Ph**-H**), 6.94 (td, *J* = 7.6, 1.2 Hz, 1H, Ph**-H**), 5.11 (t, *J* = 5.6 Hz, 1H, –CH_2_-O**H**), 4.83 – 4.78 (m, 1H, –C**H**(CH_2_-OH)), 3.63 (m, 2H, –C**H**_**2**_-OH), 3.49 (dd, *J* = 10.8, 8.9 Hz, 1H, -S-C**H**H-CH(CH_2_-OH)), 3.32 – 3.29 (m, 1H, -S-C**H**H-CH(CH_2_-OH)).

^13^C NMR (800 MHz, DMSO-*d*_6_) *δ* 171.05, 158.41, 133.21, 130.32, 119.10, 116.70, 115.82, 77.83, 62.32, 32.58.

**HR-ESI-MS:** *m*/*z* = 210.0580 [M + H]^+^ (calc. C_10_H_11_NO_2_S = 210.0583, Δppm = 1.4).

1D- and 2D-NMR spectra of aerugine are shown in Fig S2 and spectral data 1.

Aeruginol


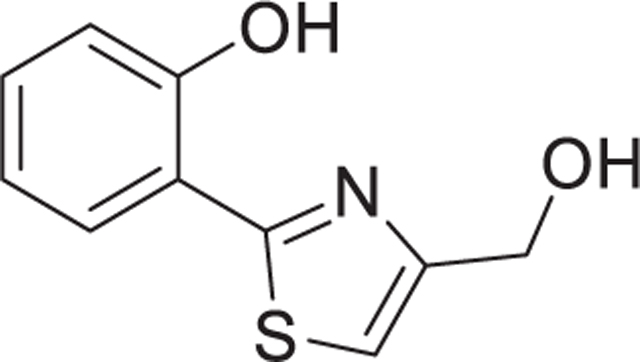


^1^H NMR (800 MHz, DMSO-*d*_6_) *δ* 11.65 (s, 1H, Ph-O**H**), 8.01 – 7.95 (m, 1H, Ph**-H**), 7.44 (s, 1H, -S-C**H** = C(CH_2_-OH)), 7.28 (t, *J* = 7.6 Hz, 1H, Ph**-H**), 7.01 (d, *J* = 8.2 Hz, 1H, Ph**-H**), 6.90 (t, *J* = 7.4 Hz, 1H, Ph**-H**), 5.38 (s, 1H, –CH_2_-O**H**), 4.62 (s, 2H, –C**H**_**2**_-OH).

^13^C NMR (800 MHz, DMSO-*d*_6_) *δ* 164.41, 156.55, 155.79, 130.95, 127.24, 119.05, 118.62, 116.84, 114.54, 59.62.

**HR-ESI-MS:** *m*/*z* = 208.0429 [M + H]^+^ (calc. C_10_H_9_NO_2_S = 208.0427, Δppm = 1.0).

1D- and 2D-NMR spectral data of aeruginol are shown in the supplementary data.

### 2.15. Synthesis of aerugine

The synthetic steps for aerugine were described by S. Noel *et al.* (Noel et al., 2011) and A. Zamri *et al.* (Zamri and Abdallah, 2000) which we used in the following synthetic route:

#### 2.15.1. Synthesis of 2-(2-hydroxyphenyl)-4,5-dihydrothiazole-4-carboxylic acid (1a)

2-Hydroxybenzonitrile (500 mg, 4.2 mmol) was dissolved in MeOH (18.7 ml) and L-cysteine hydrochloride (1.475 g, 8.4 mmol) was added. Then, 0.1 M phosphate buffer was added (pH = 6.4, 18.7 ml). The solution was adjusted to pH =6.4 again with dry K_2_CO_3_ and was stirred for 12 h at 60 °C. The white precipitate was filtered off and the flow-through was concentrated. After concentration, the residual solution as diluted with H_2_O (20 ml). The pH was adjusted to 2 by addition of solid citric acid and the cloudy solution extracted with CH_2_Cl_2_ (DCM; 3x). The combined organic layers were dried over MgSO_4_ and the solvent evaporated to yield the pure compound (738 mg, **79%**).

^1^H NMR (400 MHz, acetone-*d*_6_) *δ* 12.39 (s, 1H, –COO**H**), 11.56 (s, 1H, Ph-O**H**), 7.50 – 7.41 (m, 2H, Ph**-H**), 7.00 – 6.92 (m, 2H, Ph**-H**), 8.0 Hz, 1H, –C**H**(COOH)), 5.55 (dd, *J* = 9.2 Hz, 1H, –C**H**(COOH)), 3.81 – 3.73 (m, 2H, -S-C**H**_**2**_-CH(COOH)).

^13^C NMR (400 MHz, acetone-*d*_6_) *δ* 174.49, 171.34, 160.12, 134.43, 131.51, 119.88, 117.87, 116.86, 77.56, 34.14.

#### 2.15.2. Synthesis of methyl 2-(2-hydroxyphenyl)-4,5-dihydrothiazole-4-carboxylate (1b)

Acetylchloride (516 μl, 7.3 mmol) was dissolved in MeOH (4.0 ml) at 0 °C. 2-(2-hydroxyphenyl)-4,5-dihydrothiazole-4-carboxylic acid (**1a**, 300 mg, 1.3 mmol) was added and the solution was heated to 50 °C for 24 h. The solvent was evaporated and ethyl acetate (10 ml) was added. The organic phase was washed twice with H_2_O (2 × 10 ml) and brine. The organic layer was dried over MgSO_4_ and the solvent evaporated to yield the pure compound (263 mg, **82%**).

^1^H NMR (400 MHz, MeOD) *δ* 7.46 (dd, *J* = 7.8, 1.6 Hz, 1H, Ph**-H**), 7.43 – 7.35 (m, 1H, Ph**-H**), 6.99 – 6.87 (m, 2H, Ph**-H**), 5.50 – 5.42 (m, 1H, –C**H**(COOCH_3_)), 3.82 (s, 3H, –COOC**H**_**3**_), 3.70 – 3.66 (m, 2H, -S-C**H**_**2**_-C(COOCH_3_)).

^13^C NMR (400 MHz, MeOD) *δ* 175.51, 172.25, 160.23, 134.64, 131.81, 120.17, 118.03, 117.23, 78.05, 53.24, 34.42.

#### 2.15.3. Synthesis of aerugine (1)

To 1 ml of tetrahydrofuran was added methyl 2-(2-hydroxyphenyl)-4,5-dihydrothiazole-4-carboxylate (**1b**, 36.5 mg, 0.16 mmol) and NaBH_4_ (16.2 mg, 0.43 mmol). The solution was stirred for 15 min until it turned yellow. 45 μl H_2_O were added dropwise and stirring was continued for 10 min. Then, 635 μl H_2_O were added and the solution was extracted with diethyl ether (3 × 1 ml). The organic layer was washed with brine and was dried over Na_2_SO_4_. The solvent was evaporated and the residue was purified with semipreparative HPLC with a normal phase Si-column with Si Reprosil 100 (250 × 10 mm, 5 μm, Dr. Maisch). The solvents were: A = DCM, B = MeOH. Gradient: isocratic 10% B. At 5 min, aerugine eluted as pure compound (7.8 mg, **23%**).

^1^H NMR (400 MHz, MeOD) *δ* 7.43 (dd, *J* = 7.8, 1.7 Hz, 1H, Ph-**H**), 7.35 (ddd, *J* = 8.9, 7.2, 1.6 Hz, 1H, Ph-**H**), 6.93 (dd, *J* = 8.4, 1.2 Hz, 1H, Ph-**H**), 6.89 (td, *J* = 7.5, 1.2 Hz, 1H, Ph-**H**), 4.89 – 4.77 (m, 1H, –C**H** (CH_2_-OH)), 3.83 – 3.77 (m, 2H, –C**H**_**2**_-OH), 3.48 (dd, *J* = 11.0, 8.7 Hz, 1H, -S-C**H**H-CH(CH_2_-OH)), 3.33 (d, *J* = 3.1 Hz, 1H, -S-C**H**H-CH(CH_2_-OH)).

^13^C NMR (400 MHz, MeOD) *δ* 173.58, 160.20, 134.06, 131.60, 119.95, 117.81, 117.58, 79.51, 64.23, 33.70.

**ESI-MS:** *m*/*z* = 210.03 (calc. 210.06).

### 2.16. Synthesis of aeruginol

Aeruginol was synthesized by reduction of IQS according to Fig S3.

#### 2.16.1. Synthesis of IQS

The synthesis was performed according to F. Shang *et al.* (Shang et al., 2014). In short, 2-bromothiazole-4-carbaldehyde (115 mg, 0.6 mmol), LiCl (53.8 mg, 1.27 mmol) and Pd(PPh_3_)_4_ (30 mg, 0.026 mmol) were added to a dry Schlenk tube under N_2_. Then, 2-hydroxylphenylboronic acid pinacol ester (158 mg, 0.72 mmol), 4 ml dry toluene, 4 ml ethanol and 2 ml 1 M aq. Na_2_CO_3_ were added. The mixture was heated to 110 °C and stirred for 23 h. The mixture was concentrated *in vacuo* and diluted with 20 ml CH_2_Cl_2_, washed with 20 ml H_2_O and 20 ml brine. The organic phase was dried over MgSO_4_ and the solvent evaporated. Automated flash chromatography with 1:1 hexane:EA yielded a semi-pure product, which was further purified with semipreparative HPLC with a normal phase Si-column with Si Reprosil 100 (250 × 10 mm, 5 μm, Dr. Maisch). The solvents were: A = EA, B = hexane. Gradient: T_0min_ = 30% B, T_1min_ = 30% B, T_15min_ = 0% B. At 4 min, IQS eluted as pure compound (1.35 mg, **1%**).

^1^H NMR (400 MHz, CDCl_3_): 11.58 (1H, br s, Ph-O**H**), 10.07 (1H, s, C (C**H**O)), 8.13 (1H, m, -S-C**H** = C(CHO)-), 7.63 (1H, m, Ph**-H**), 7.39 (1H, m, Ph**-H**), 7.12 (1H, m, Ph**-H**), 6.96 (1H, m, Ph**-H**). HR-ESI-MS: *m*/*z* = 206.0270 [M + H]^+^ (calc. 206.0270, Δppm = 0).

#### 2.16.2. Aeruginol

NaBH_4_ (0.5 mg, 0.02 mmol) was added to a solution of IQS (2.0 mg, 0.01 mmol) in 1 ml THF. The solution was stirred at room temperature for 15 min. 50 μl H_2_O were added dropwise and stirring was continued for 10 min. Then, 100 μl H_2_O were added and the solution was dried over Na_2_SO_4_. The solvent was evaporated and the reaction yielded a pure compound (1.34 mg, **67%**).

^1^H NMR (400 MHz, DMSO-*d*_6_ (400 MHz): 11.42 (1H, br s, Ph-O**H**), 7.96 (1H, d, *J* = 7.7,Ph**-H**), 7.47 (1H, s, -S-504 C**H** = C(CH2-OH)), 7.31 (1H, t, *J* = 7.7, Ph**-H**), 7.02 (1H, d, *J* = 8.3, Ph**-H**), 6.94 (1H, t, *J* = 7.5, Ph**-H**), 5.38 (1H, br s, –CH2-O**H**), 4.62 (2H, s, –C**H**_**2**_-OH).

### 2.17. Data analysis

Unless otherwise stated, all data are expressed as means ± SEM of at least three biological replicates, each of them based on three technical replicates. The methods for statistical analysis are indicated in the respective figure legends.

## 3. Results and discussion

### 3.1. Purification of neurotoxic metabolites from Streptomyces venezuelae extracts

**Fig. 1.**
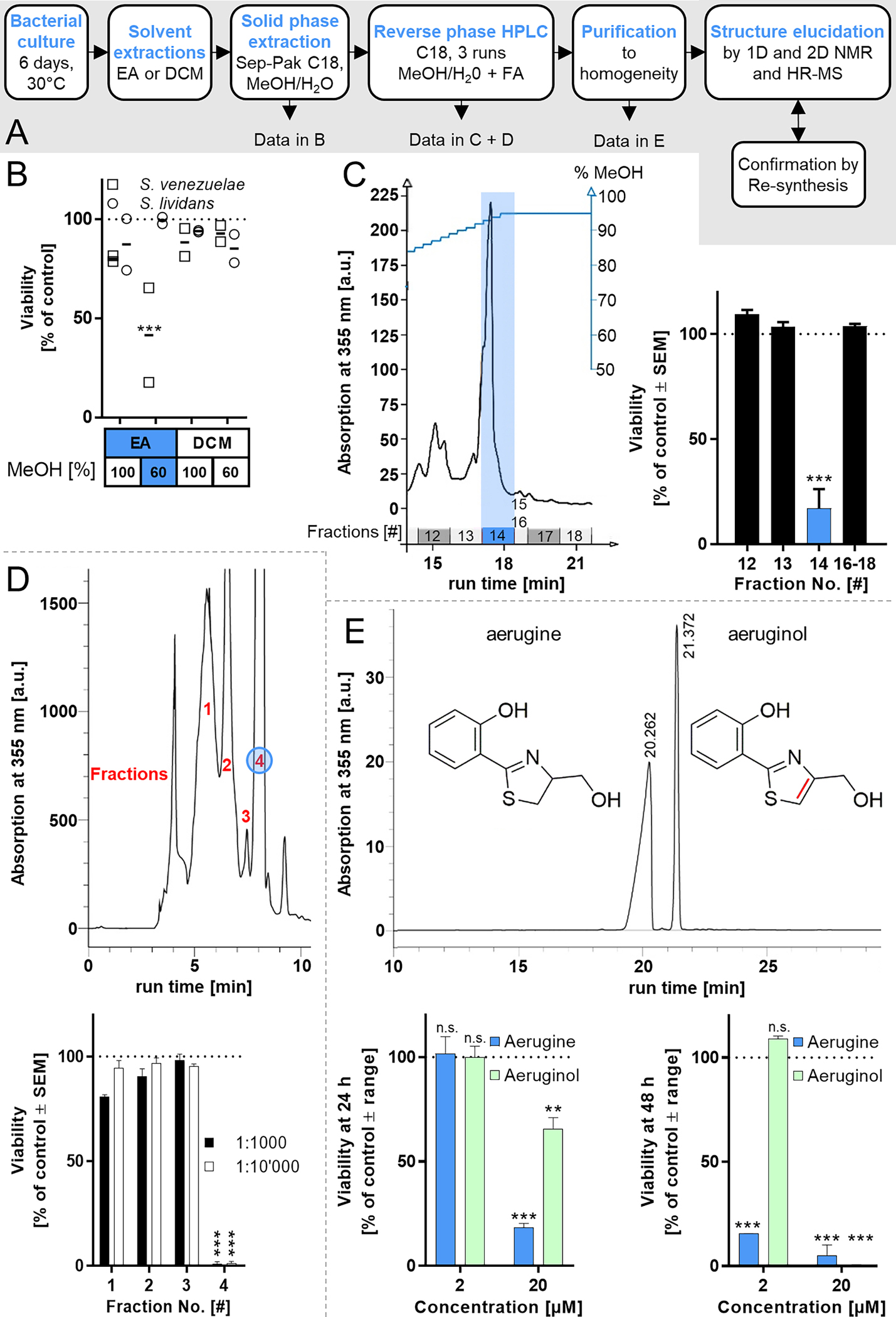
Purification of neurotoxic small molecules from *Streptomyces* bacterial strains. A Workflow of metabolite identification from bacterial extracts. Subfigures that provide activity data on the respective purification stages are indicated. Information on the purification procedures is given in the subfigure paragraphs below. B-E The neurotoxicity of extracts, fractions and pure compounds was tested on human dopaminergic neurons. After 6 days of differentiation (d6), cultures of LUHMES neurons were exposed to bacterial fractions. The cell viability was assessed by calcein-AM & H-33342 staining, automated fluorescence microscopy and quantification by an image processing algorithm. B Cultures of *Streptomyces venezuelae* and *S. lividans* were extracted with ethyl acetate (EA) or dichloromethane (DCM). The crude extracts were fractionated *via* Sep-Pak C18 and eluted using a stepwise gradient with methanol and water (MeOH = 60% or 100%). Dried and DMSO-reconstituted fractions were tested for effects on neuronal viability. Data from at least two fully independent runs. Viability data was obtained after 24 h of exposure. C The EA/60% MeOH extract of *S. venezuelae* was further fractionated by reverse phase HPLC (C18 column, MeOH gradient). The collected fractions are indicated in the absorption spectrum (a part of the spectrum is shown, the full spectrum is given in Fig S1; the MeOH gradient started at 50%). Fractions were dried, taken up in DMSO and added to neurons for 48 h (final dilution 1:1000). Viability data are means ± SEM (n = 3). D Fraction 14 of the previous step was further purified by several reverse phase HPLC (C-18) runs in acetonitrile/water eluent. Absorption peaks were collected in fractions as depicted (red numbers in the spectrum). They were tested for their effect on neuronal viability (24 h, at 1:1000 and 1:10000 dilutions). E Two single substances (aerugine and aeruginol) of fraction 4 of the previous purification step were separated and purified to homogeneity. The chemical structures (elucidation details in Fig S2 and spectral data 1) are displayed next to their respective peaks in the elution trail diagram. Note the additional double bond in the thiazolidine ring of aeruginol. The two compounds were assessed in two separate experiments for their effect on neuronal viability after incubation times of 24 h and 48 h. All statistical analyses were performed using a one-way ANOVA with Dunnett’s multiple comparisons test (** = p < 0.005, *** = p < 0.0001).

Previously, the existence of a microbial metabolite with specific neurotoxic properties had been proposed, but its structural identification remained elusive (Caldwell et al., 2018; Caldwell et al., 2009). To fill this knowledge gap, we sequentially perfomed an activity-guided fractionation of bacterial lysates, using a neurotoxicity test as bioassay (Fig. 1a). As a starting point, we used cultures of *S. venezuelae* (presumed to be neurotoxic) and cultures of *S. lividans* (phylogenetically related, yet deemed non-toxic) (Caldwell et al., 2009)) that were extracted with different solvent combinations and subsequently pre-fractionated over reverse-phase C18 Sep-Pak columns. One of the *S. venezuelae* fractions killed >50% of a DAergic human neuronal cultures (produced from LUHMES cells), while a similarly-prepared extract of *S. lividans* was non-cytotoxic (Fig. 1b).

The bioactive lysate was purified by HPLC runs, until a fraction was obtained that still showed bioactivity (killing neurons) when diluted 10000-fold (Fig. 1c,d; Fig S1). From this fraction, two compounds were each purified to homogeneity by multiple HPLC runs and the structure identified by 1D and 2D NMR spectroscopy in combination with mass spectrometry (Fig S2). Both compounds, aerugine (209.27 g/mol) and aeruginol (207.25 g/mol) were confirmed to be cytotoxic to DAergic neurons in a time- and concentration-dependent manner (Fig. 1e). As aerugine was more potent (low μM range), further work concentrated on this metabolite. Its structure was confirmed by re-synthesis (Fig S3), and an overview of its physicochemical properties was obtained. The compound was predicted to reach intracellular compartments and to cross the blood-brain barrier without need for transporters (Fig S4).

### 3.2. Biosynthetic pathway of aerugine

*S. venezuelae* is a soil-living bacterium, and its production of a neurotoxic metabolite may be of little relevance to humans or most other vertebrates. However, genetic elements required for synthesis of secondary metabolites may be wide-spread amongst bacteria. The gut microbiome produces a large variety of metabolites that may influence physiology and pathology of the local and central nervous systems (Baldini et al., 2020; De Miranda et al., 2022; Dodd et al., 2017; Donia and Fischbach, 2015; Nicolas and Nolan, 2022; Sampson et al., 2016; Swann et al., 2020). We used here published literature and databases to identify the likely biosynthetic pathway of aerugine, and to check whether the responsible genetic machinery may also be found in human pathogens or the intestinal flora.

Indeed, the compound identified here, has been described earlier as a byproduct of watasemycin biosynthesis. Moreover, the biosynthetic relevance of the underlying genetic machinery has been proven by detection of aerugine in cultures of *S. coelicolor* M1152, which were engineered to express the watasemycin gene cluster (Inahashi et al., 2017). Our analysis of the gene sequence of *S. venezuelae* (ATCC 10712) used in this study confirmed the presence of this non-ribosomal peptide synthetase (NRPS) biosynthetic gene cluster (Fig S5). The synthesis uses the building blocks salicylic acid *plus* cysteine. Aerugine is likely to be generated from a watasemycin precursor by a non-enzymatic hydrolytic step. A highly homologous biosynthetic machinery is used by *Pseudomonas aeruginosa* to generate the siderophore pyochelin. Aerugine has been previously described as by-product of pyochelin biosynthesis (Kaplan et al., 2021). In enterobacteriae, the identical precursor is further modified via a mixed NRPS – polyketide synthetase (PKS) involving condensation of a malonyl-CoA and one additional cysteine to the full length product yersiniabactin (Miller et al., 2002). Siderophores of the watasemycin-pyocheline-yersiniabactin family are widespread and generated by biosynthetic machineries that likely enable the production of aerugine as shunt-product. Several producers are found in the human intestine (*Pseudomonas* sp., *E. coli*, *Yersinia* sp., *Salmonella* sp. etc.) (Fig S6). Within the compound family, aerugine is most-closely related to pulicatins. The latter compounds are methylated in position five of the thiazoline ring. Very little information is available on human-relevant bioactivities. The only suggested target for aerugine is the 5HT_2B_ receptor, from which it displaced radioactive lysergic acid diethylamide at low μM concentrations. However, functional data are not available to our knowledge (Lin et al., 2017).

### 3.3. Activity confirmation and primary characterization

**Fig. 2.**
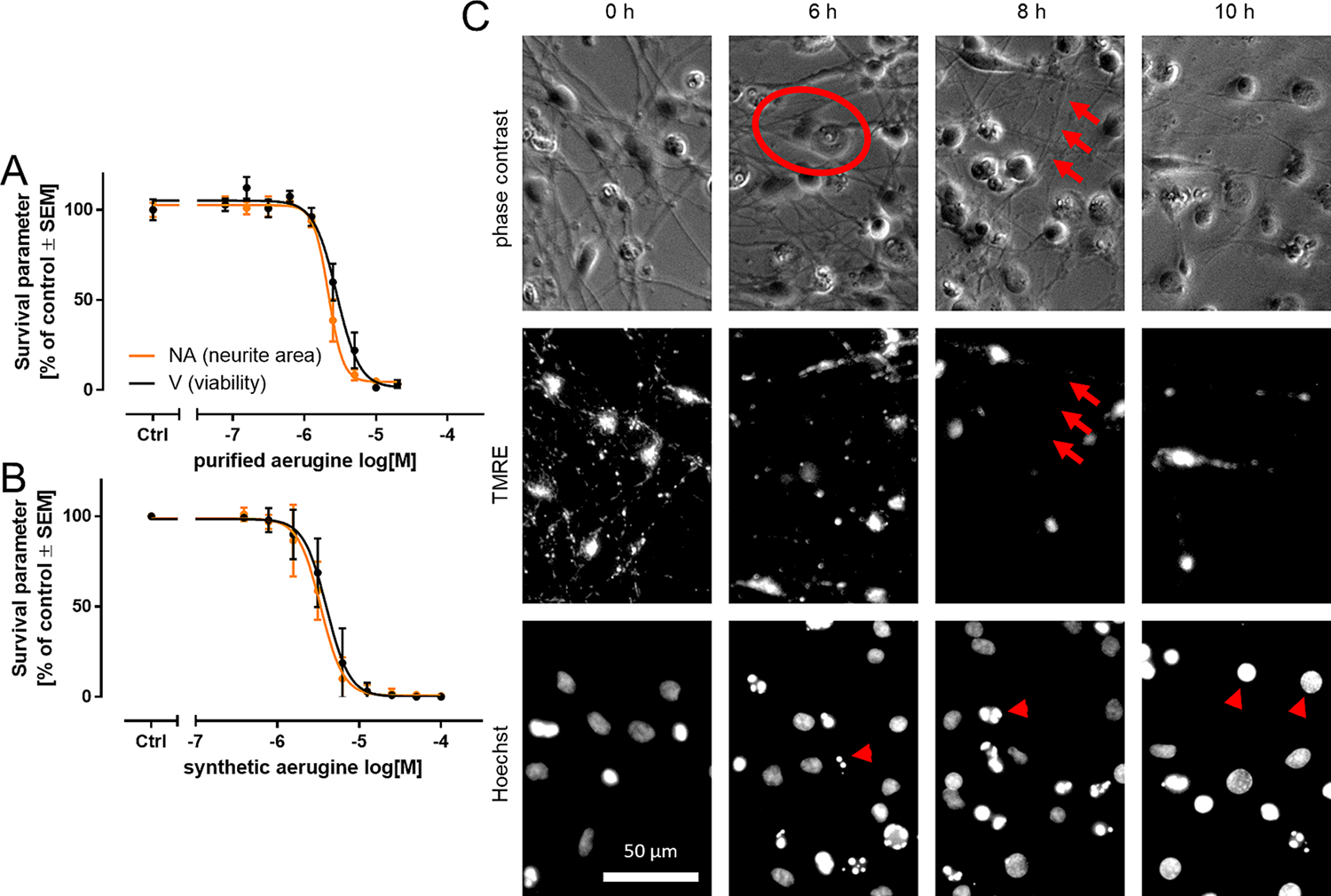
Degeneration of human dopaminergic neurons exposed to aerugine. Cultures of differentiated LUHMES neurons were used on d6 for cytotoxicity testing. Cells were exposed to serial dilutions of (A) aerugine purified from bacterial extracts and (B) synthetic aerugine. The cell viability (V) and neurite area (NA) were assessed 24 h later by calcein-AM & H-33342 staining and high content imaging. Data are presented as means ± SEM of three biological replicates. C LUHMES neurons (d6) were treated with 10 μM aerugine. After the indicated incubation times, they were stained with H-33342 & the mitochondrial membrane potential indicator tetramethylrhodamine ethyl ester (TMRE). Representative images after exposure to aerugine are shown. Cells reacted similarly to purified aerugine. The scale bar represents 50 μm. A flattened cell is circled. Arrows indicate an intact neurite without functional mitochondria. Arrow heads point to features of apoptotic nuclear condensation. Enlarged details are shown in Fig S8.

After having obtained information on aerugine biosynthesis, we compared the bioactivity of purified preparations and pure synthetic product. The cytotoxic potency of extracted compound was similar to that of aerugine prepared by chemical synthesis (Fig. 2a,b), and the EC50 value was 3.1 ± 0.7 μM. Accordingly, all further experiments were performed with synthetic aerugine.

The standard assay used to evaluate toxic effects on neuronal cultures is based on single cell staining with a live cell dye (calcein). This allows also the quantification of potential effects on neurite structures (Delp et al., 2019; Stiegler et al., 2011). In the 24 h assay setup, aerugine affected neurites to the same extent and at similar concentrations as cell bodies (Fig. 2a,b). Biochemical viability assays that measure the average of a whole culture well (lactate dehydrogenase (LDH)-release and resazurin reduction tests) confirmed the single cell data, although with slightly less sensitivity (Fig S7).

To obtain additional information on the death process triggered by aerugine, we also examined earlier time points after exposure to a fixed concentration of 10 μM. Semi-quantitative information was obtained from phase contrast images, by staining of energized mitochondria with the potential-sensitive dye tetramethylrhodamine ethyl ester (TMRE) and by visualization of the chromatin structure with the DNA-intercalating dye H-33342. After 6 h exposure, clear morphological changes became obvious. While the neurite structure was well-preserved, some of the cell bodies flattened in a way that the nucleus became visible in phase contrast. The normally round/dome-like cells took a flattened “fried egg” structure. In parallel, the DNA condensed and fragmented into small, round and highly-fluorescent spheres, a typical hallmark of apoptosis. Cells with apoptotic nuclei had lost their TMRE stain. At 8 h, more cell bodies looked flattened (“unhealthy”) and the neurites were thinning. Even within structurally intact neurites (no blebbing, sharp contrast) the mitochondrial membrane potential was lost (absence of TMRE staining). At 10 h, the majority of neurites was lost, and most nuclei were condensed (Fig. 2c; Fig S8). This sequence of events is typical of neuronal apoptosis, *e.g.* under mild excitotoxic conditions, or after exposure to proteasome inhibitors or cytoskeletal poisons (Gutbier et al., 2018b; Leist et al., 1997; Volbracht et al., 2001; Volbracht et al., 1999). The overall kinetics of cell death was rather fast, compared to many other toxicants. This suggests that aerugine directly affects one of the cell biological processes essential for survival. To obtain a more quantitative confirmation of the rapid process, cells were stained with calcein-acetoxymethyl ester and propidium iodide (PI) and observed over time. The staining of nuclei (indication of plasma membrane permeability) by PI was quantified by an imaging algorithm. By 6 h, dead cells increased by 17 ± 6%, by 8 h by 25 ± 5%, and by 10 h, > 95% of the cell nuclei were positive. This confirms the fast cell death process triggered by aerugine (10 μM) in neurons.

We explored here a potential mitochondrial toxicity of aerugine, as mitochondria are one of the most obvious vital structures in neurons, and a large body of evidence points to the role of mitochondrial impairment especially in toxicity to DAergic neuronal death (Delp et al., 2019; Huang et al., 2022; Schildknecht et al., 2017). Initially, we evaluated the effect of aerugine on mitochondrial respiration. The oxygen consumption rate (OCR) in the presence of aerugine was not different from control conditions, even when tested under various metabolic conditions (Fig S9a). Also, when various functional parameters (*e.g.,* basal respiration or ATP production) were calculated, no significant difference was observed (Fig S9b). To confirm these data, we made use of the fact that LUHMES neurons can be made extremely dependent on mitochondrial respiration by exchange of glucose in the medium to galactose. Under such conditions, mitochondrial inhibitors can be detected with up to 1000-fold increased sensitivity (Delp et al., 2019). However, also under this condition, no increased aerugine toxicity was observed (Fig S9c,d). It seems therefore unlikely that aerugine directly affects mitochondrial respiration. More efforts are required in the future to identify the cell biological target process of this neurotoxicant. The clear effects of aerugine on mitochondrial membrane potential during cell death are likely to be caused indirectly.

### 3.4. Specificity of aerugine neurotoxicity

Although aerugine was identified here based on its activity as neurotoxic (Fig S10a), we could not assure that our findings were neuro-specific. It was also unclear, whether we witnessed an unspecific effect of substituted phenolic compounds or a specific event triggered only by aerugine and very closely-related structures. To address this latter point, we compared aerugine to three structural analogs that were available to us. When the aliphatic OH-group of aerugine was oxidized to an aldehyde (dhIQS) or a carboxylic acid (DHAA), all toxicity was lost (Fig S10b,c). This indicates that there is a specific structural requirement for aerugine to be neurotoxic. The aldehyde of aeruginol (IQS) killed DAergic neurons within 24 h in the 10–25 μM concentration range, *i.e.* it was about equipotent with aeruginol (Fig S10d). This indicates that some close analogs of aerugine may also be neurotoxic. Further quantitative structure-activity relationship (QSAR) studies are required to better understand the toxicological potential and mechanism of the whole watasemycin-aerugine family.

**Fig. 3.**
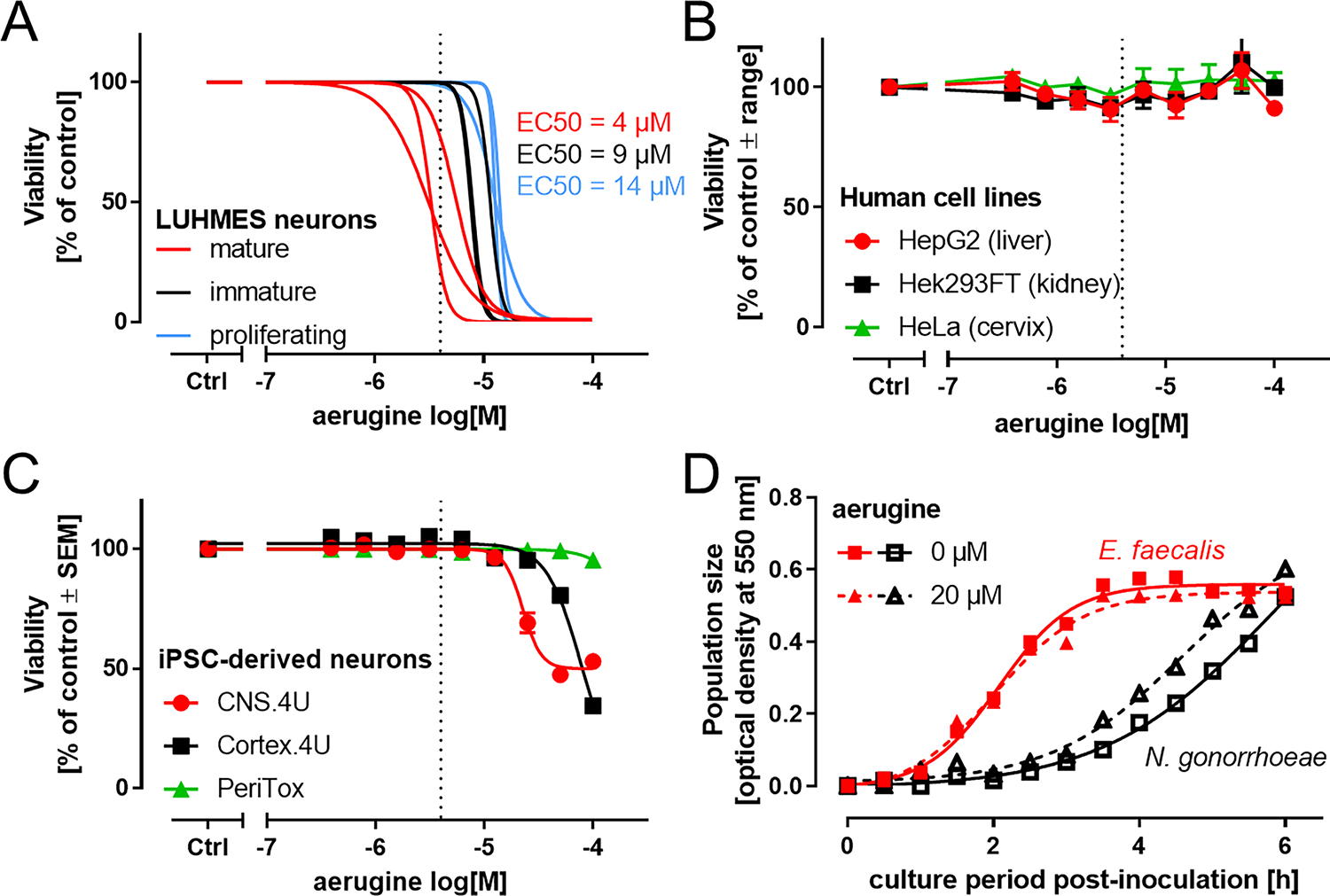
Cell type specificity of aerugine cytotoxicity. A Cultures of LUHMES neurons were used on d0 (proliferating, blue), d2 (immature, black) or d6 (mature, red) of differentiation for cytotoxicity testing. Cells were exposed to nine serial dilutions of synthetic aerugine. The cell viability was assessed 24 h later by calcein-AM & H-33342 staining and high content imaging. Data are presented as sigmoidal curve fits of data points from each different experiment (averaged data points are displayed in Fig S11). The half-maximal cytotoxic potency (EC50) is displayed for the three maturity stages. Dotted lines at 4 μM indicate the average EC50 for mature LUHMES. B Three widely-used human cell lines were cultured as suggested by the supplier/cell bank and used for experiments in 96-well plates. They were treated for 72 h with synthetic aerugine. The overall cell viability was assessed by measuring resazurin reduction in technical triplicates. Data are presented as means ± range of two biological replicates (different experiments). C Three iPSC-derived neuronal cell types were treated for 24 h with synthetic aerugine. The cell viability was assessed 24 h later by calcein-AM & H-33342 staining and high content imaging. Data are presented as means ± SEM of three biological replicates (different experiments). D Lack of cytotoxic or bacteriostatic effects of aerugine (20 μM) on exemplary gram-positive and gram-negative microbes: The growth characteristics of *Enterococcus faecalis* (gram-positive) and *Neisseria gonorrhoeae* MS11 (gram-negative) were followed during the log growth phase in their respective culture media. Data are from one biological replicate.

To investigate the cell type specificity, we first compared LUHMES cells of different maturity. We found that their sensitivity to aerugine increases as they differentiate. The EC50 values for proliferating, immature and mature cells were 13 ± 0.2 μM, 9.3 ± 1.1 μM, and 4.1 ± 0.8 μM (Fig. 3a; Fig S11). Thus, even within the same cell line, the neuronal maturity state increased aerugine sensitivity approximately three-fold. When we examined aerugine toxicity to other commonly used human cell lines (HepG2 – liver-derived, Hek293FT – kidney-derived, HeLa – cervix-derived), no toxicity was observed, even with a prolonged exposure of 72 h (Fig. 3b). To confirm the toxicity to neurons, we obtained various human stem cell-derived cultures. Mixed central nervous system neurons were clearly affected by aerugine (but at higher concentrations), while peripheral nervous system cells showed no effect at concentrations up to 100 μM. Cortical neurons (also representative for the central nervous system, but not containing DAergic neurons) were also sensitive to aerugine, but at about 50x higher concentrations than DA neurons (mature LUHMES) (Fig. 3c). Finally, we also addressed the toxicity to prokaryotes. We used *Enterococcus* and *Neisseria gonorrhoeae* as representatives of gram-negative and gram-positive bacteria. They were not affected in viability or growth at 20 μM aerugine, a concentration that would kill all mature DA neurons (Fig. 3d). We conclude from these data that aerugine is not a general, unspecific cytotoxicant. It rather has a considerable specificity for DA neurons.

### 3.5. Pharmacological modulation of aerugine toxicity

**Fig. 4.**
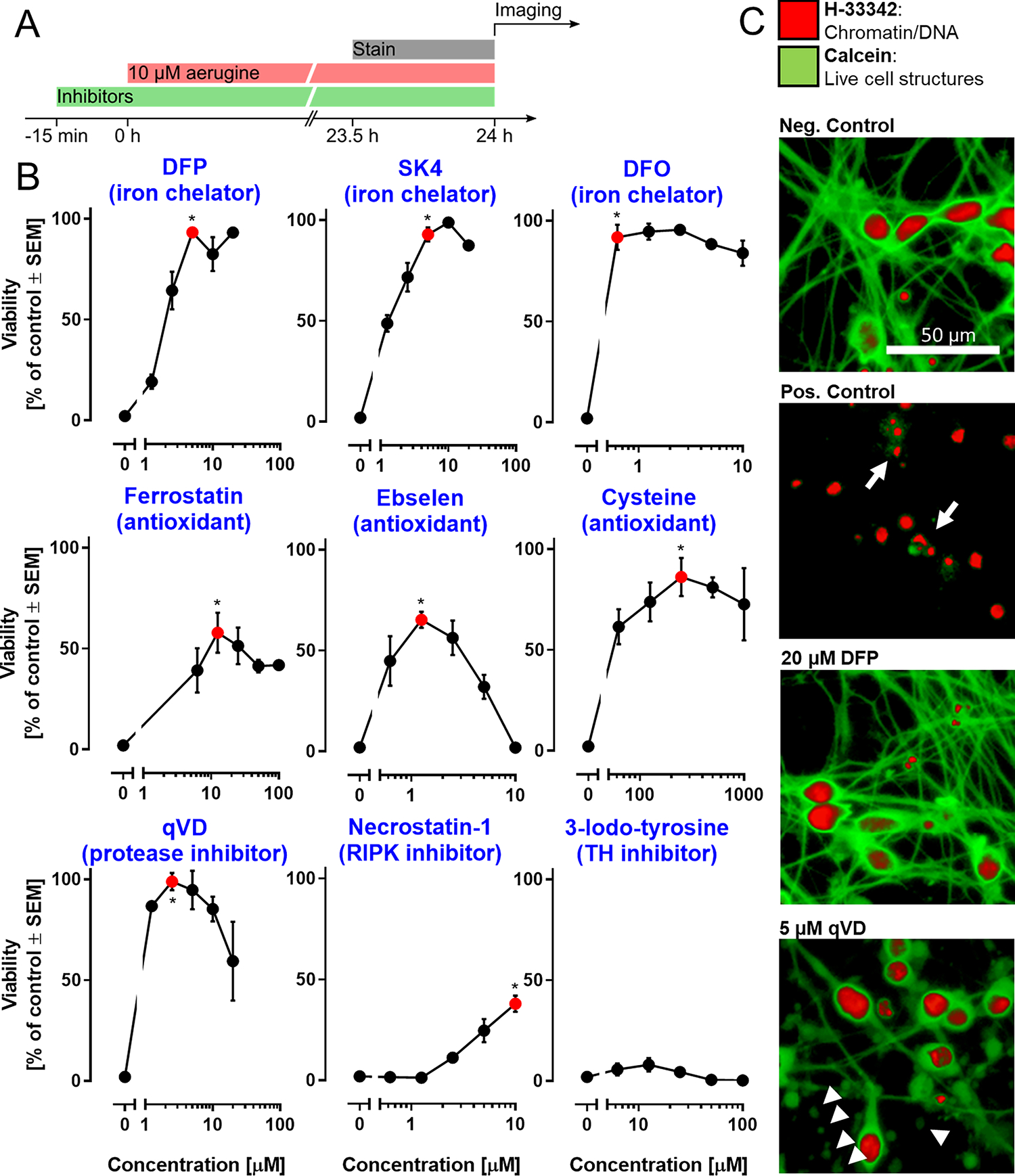
Modulation of aerugine-induced neurodegeneration by mechanistic inhibitors. LUHMES cultures (d6) were used under standard conditions to assess the cytotoxicity of aerugine (10 μM) in the presence of various agents known to interfere with cell death pathways. A Treatment scheme: the respective inhibitors were applied 15 min prior to aerugine treatment (10 μM), and the viability was determined by high-content imaging after 24 h. B Inhibitors were used at different concentrations, as indicated. The main biochemical activity of each agent is indicated in brackets. Data are means ± SEM of at least three biological replicates with 3 technical replicates each. DFP = deferiprone; DFO = deferoxamine; qVD = caspase inhibitor; RIPK = receptor-interacting serine/threonine-protein kinase. For statistical analysis, the lowest concentration of inhibitor that produced the maximal protective effect (red) was compared to the treatment with aerugine alone using a two-tailed *t*-test (*: p = 0.01). C Exemplary pictures of LUHMES cultures after 24 h treatment: negative control (=DMSO), positive control (=10 μM aerugine), DFP (=20 μM DFP + 10 μM aerugine) and qVD (=5 μM qVD + 10 μM aerugine). Arrows indicate cells with a broken cell membrane (calcein-negative) and condensed nuclei (small H-33342-positive area). Arrow heads indicate disintegrating neurites (chains of calcein-positive blebs). Enlarged details are given in Fig S12.

A typical feature of specific toxicants is that their effects on cell health/survival can be prevented by inhibitors or modifiers of downstream pathways that are affected. We used here a panel of nine such modulators and studied their effect on the neurotoxicity of aerugine (Fig. 4a). Three iron chelators (used in the low μM range) completely prevented cell death (Fig. 4b). Under these conditions, not just the cell bodies survived, but also the neurite structure was well-preserved (Fig. 4c). Rescue by iron chelators may point to a ferroptotic death mechanism (Lin et al., 2022), but iron may also play a role in other types of cell death, such as apoptosis or lysosomal death (Bogdan et al., 2016; Chen et al., 2019; Terman and Kurz, 2013; Whitnall and Richardson, 2006). Especially, DAergic neuronal death has many associations with iron overload (Devos et al., 2014; Gutbier et al., 2020; Riederer et al., 2021; Whitnall and Richardson, 2006).

Iron is a key player in oxidative stress pathways. Consistent with this, three different types of antioxidants (ferrostatin: radical scavenging; ebselen: SOD mimetic; cysteine: glutathione precursor) partially (maximally 60–90%) prevented aerugine neurotoxicity. The pronounced rescue by cysteine may be interpreted as pointing towards ferroptosis (Morris et al., 2018). However, cysteine and related compounds (*e.g.*, *N*-acetylcysteine) are also anti-apoptotic. For instance, LUHMES cells driven into apoptosis by proteasome inhibitors, can be rescued by cysteine (Gutbier et al., 2018b; Suciu et al., 2023).

A role of apoptosis in aerugine neurotoxicity is suggested by the protective effect of the protease inhibitor qVD. At optimal concentrations (2–4 μM) cell death was completely prevented (rescue of all cell somata). However, damage to the neurites still occurred in the presence of qVD. This set of observation is consistent with a role of apoptotic caspase activation in aerugine toxicity (Fig. 4b,c; Fig S12). In contrast, the necroptosis-inhibitor necrostatin-1 only slightly attenuated cell death. The tyrosine hydroxylase inhibitor 3-iodo-tyrosine had no effect at all. The latter finding suggests that DA autoxidation, a process sometimes associated with DAergic neurotoxicity, plays no major role. While more mechanisms need to be investigated to find the target of aerugine, our pharmacologic intervention data suggest that aerugine triggers defined death-associated pathways, and inhibition of these pathways abolishes neurotoxicity, also in the continued presence of the toxicant.

### 3.6. In vivo toxicity of aerugine to C. elegans dopaminergic neurons

Our study was triggered by the observation that metabolite extracts of *S. venezuelae* caused selective DAergic neurotoxicity in the nematode *C. elegans* (Caldwell et al., 2009). Here, we isolated aerugine as human DAergic neurotoxicant, showed its cell type specificity and the triggering of defined death pathways (inhibited by pharmacological intervention). However, experimental proof is required that aerugine indeed reproduces the effects of bacterial extracts in a viable model, such as *C. elegans.* Moreover, studies using a complex life organism are an important confirmation of the postulated cell type specificity of aerugine-induced neurotoxicity.

**Fig. 5.**
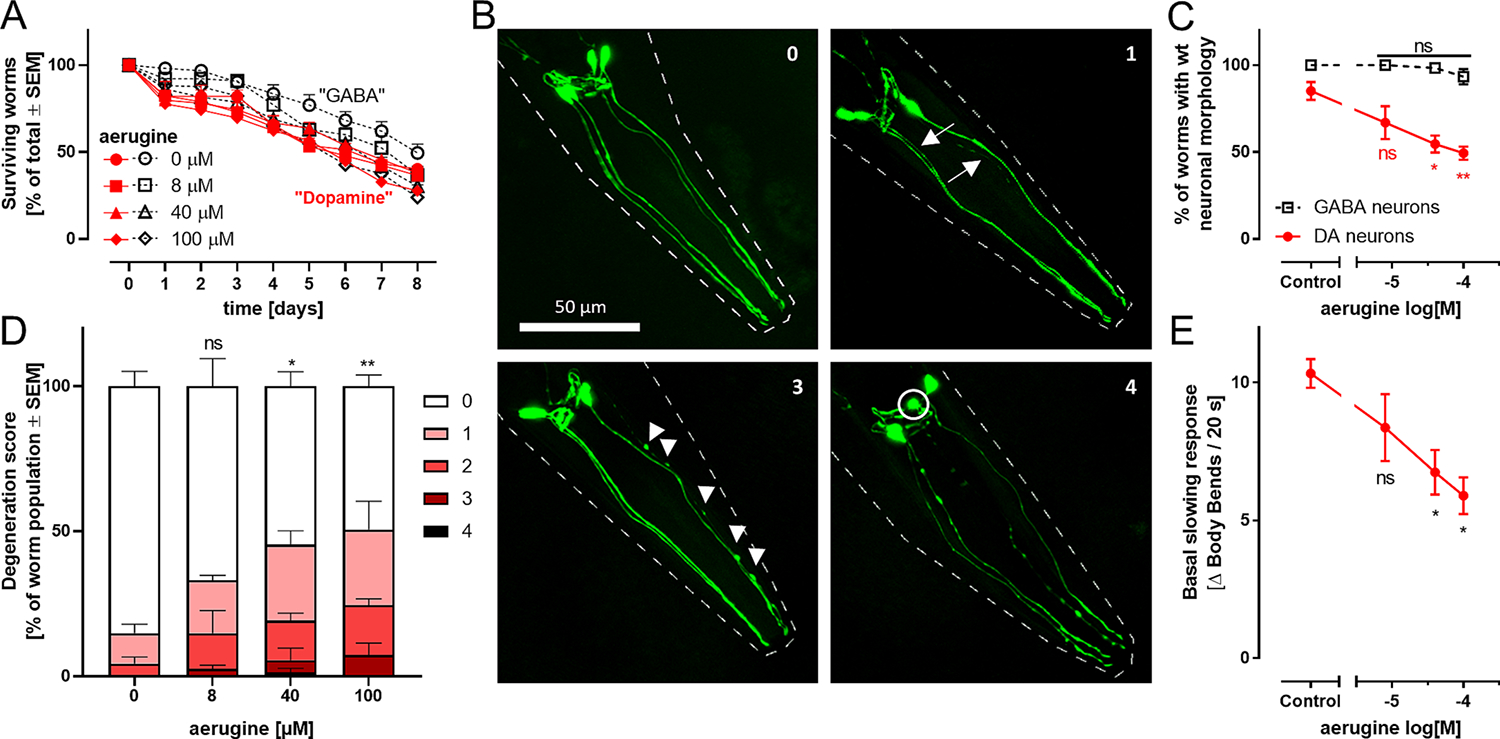
Specific dopaminergic neurodegeneration triggered by aerugine in *C. elegans*. A The *C. elegans* worm strains BY200 (containing fluorescently labelled dopamine neurons; label “Dopamine”) and Punc-25 (containing fluorescently labelled γ-aminobutyric acid neurons, label “GABA”) were used at L4 larval stage for an eight day survival test. They were exposed to different aerugine concentrations and counted every 24 h. Data are based on 40–60 worms per group. Differences on a given day were analyzed by ANOVA and found to be non-significant. Two-way ANOVA (time × treatment) indicated a significant trend of decreased survival over time (independent of treatment or genotype). B L4 larval stage worms of both strains were treated for 2 days with aerugine. The GFP-labelled neurons of 20–30 worms per condition were analyzed and scored for specific neurodegeneration. Each worm was assigned a degeneration score (0 for wild type and 1–4 for increasing severity). Representative images illustrating neurodegeneration scores (indicated by numbers in the upper right corner): The pictures show the 4 cephalic dopaminergic neurons in green. The worm outline is indicated by dashed white lines. Arrows highlight thinning dendrites. Arrow heads point out blebs (irregular protrusions of the dendrites). Degenerating cell bodies are circled. Degeneration score 2 looks similar to 3 but has a maximum of 4 blebs. These scores were assessed to quantify the dopaminergic neurodegeneration caused be aerugine *in vivo*. C Worms with wild type morphology (score 0) were counted. For statistical analysis, a one-way ANOVA with Dunnett’s multiple comparisons test was performed (*: p < 0.05; **: p < 0.01 for difference of aerugine groups vs untreated controls). D The degeneration scores exemplified in B were quantified. The data for DAergic neurons is shown. The data for GABAergic neurons can be found in Fig S13. E The “basal slowing response” was assessed as proxy for the functionality of the worms’ dopaminergic system. It was measured in L4 larval stage BY200 worms treated for 2 days with aerugine. As the slowing response is triggered by food, the body bends per 20 s in the absence and presence of food were counted and the difference (Δ) was calculated as experimental readout. For statistical analysis, a one-way ANOVA with Dunnett’s multiple comparisons test was performed (*: p < 0.05).

To address this question, we used transgenic worms with green fluorescent protein (GFP) expression targeted to either DA neurons or γ-aminobutyric acid (GABA)-neurons. They were exposed to aerugine at the L4 larval stage for up to one week. In this experimental setup, the number of surviving worms declined over time. However, aerugine neither triggered death during the first 2 days (when there was little natural death), nor did it enhance/accelerate the natural decline over longer time periods (8 days) (Fig. 5a). The GFP-labelling allowed the assessment of DAergic neuron integrity in the worms (4 labelled neurons, including their neurite structure were scored) (Fig. 5b). An assessment after 48 h exposure showed a concentration-dependent degeneration of DA neurons in aerugine-exposed worms, while GABA neurons were not affected, and the worms survived (Fig. 5c,d; Fig S13 for detailed scoring). Up to 50% of the DAergic neurons lost their normal (fully) healthy phenotype, with the majority of effects being detected on the neurite integrity.

To understand the functional consequences of this morphological evaluation, we used a behavioral endpoint strongly dependent on DAergic signaling. We made use of the fact that worms, normally body-bending in their growth medium, move less dynamically, when they are exposed to feed (de Bono and Maricq, 2005; Krum et al., 2021; Sawin et al., 2000). We assessed this “basal slowing response” (BSR) in nematodes exposed to food (bacteria) in the presence or absence of aerugine. A concentration-dependent response was observed, *i.e.* the BSR was attenuated by the neurotoxicant. This indicates that the function of the DAergic system was disturbed by aerugine.

## 4. Outlook and conclusions

In summary, our study provides an entirely novel perspective on human health implications of the environmentally-produced bioactive compound aerugine. We first corroborated earlier, well-documented observations (Caldwell et al., 2009), that a microbial-derived agent can trigger pathological processes relevant to idiopathic PD. Next, we proceeded to identify the low molecular weight, stable and neurotoxic chemical postulated in previous studies to be the DAergic toxin (Caldwell et al., 2018; Caldwell et al., 2009). Knowledge on the molecular structure of aerugine allowed us to identify the corresponding biosynthetic gene cluster. An important implication of this work is that not only soil bacteria (S. *venezuelae*) may be the source of the potent neurotoxicant aerugine, but also that constituents of the normal or of the disease-associated human microbiome (*E. coli* or *P. aeruginosa*) may produce this or related compounds.

By using a panel of cell death pathway inhibitors, we identified several compounds that attenuated or even completely blunted the toxicity of aerugine. Both, the data from our study and general knowledge on the iron-chelating properties of aerugine-related compounds (see Fig S6 for structural similarity to pulicatins) are consistent with a cell death mechanism related to iron-enhanced oxidative stress. Oxidative stress plays an important role in many regulated and non-regulated death modes (Dionísio et al., 2021; Orrenius et al., 2007). Long before the predominantly oxidative types of cell death, *i.e.,* ferroptosis and oxeiptosis, have been defined as stand-alone entities (Jiang et al., 2021; Scaturro and Pichlmair, 2019), the generation of reactive oxygen species and the loss of cellular redox-buffers was known as common features of most types of dying cells. This observation covered the full cell death spectrum, ranging from necrosis to apoptosis (Tang et al., 2019).

Iron can take a key role in cellular demise, as it converts hydrogen peroxide, which has a low-reactivity to cellular structures, *via* the Fenton reaction into the highly reactive and destructive hydroxyl radicals. Low amounts of redox-reactive metals can be detrimental, as they drive the so-called “iron-enhanced Haber-Weiss cycle”. This catalytical role of free ferric and ferrous iron in the production of reactive oxygen species is well-established to occur within live cells (Kehrer, 2000) and it plays an important role in DAergic neurons and in PD (Cerasuolo et al., 2023; Gutbier et al., 2020; Lin et al., 2022). To fully appreciate the potential role of iron in cell damage, it is important to consider various cellular iron pools. The total iron concentration in brain cells is in the low mM range, but most of it is bound to ferritin in a catalytically inactive form (Philpott and Ryu, 2014; Reinert et al., 2019; Wu et al., 2023). Only nM levels are found as free iron ions in the presence of oxygen and at neutral pH. However, cells contain a substantial pool of so-called “labile iron” (Kaplan and Ward, 2013; Lv and Shang, 2018), which describes metal ions complexed to glutathione, glutaredoxin and other cellular components. Such loosely complexed iron can participate in catalytic reactions that generate reactive oxygen species. Moreover, it is well-established that certain chelators even enhance this catalytic activity (Luzzatto et al., 1995; Miller et al., 2016; Szarka et al., 2022). A compound like aerugine may thus cause iron-dependent toxicity by changing availability and complexation of cellular iron, by slightly shifting cellular iron pools or by facilitating the import of additional extracellular iron into the cytosol. Our finding of protection by several antioxidants and iron chelators would be compatible with such a mechanism. To provide further evidence, technologies to quantify distinct iron pools, their redox states and their catalytic activity will be required (Lv and Shang, 2018).

We suggest here that the postulated death mechanism should explain (to some extent) the cell type selectivity. One may question whether iron, which is present in all mammalian cell types, is likely to be a key player in the extremely specific toxicity of aerugine to DAergic neurons. However, a selectivity of iron-mediated cell death is consistent with literature data. For instance, there are large differences in ferroptosis sensitivity of various cell types. While some cells are fully resistant, neurons are a particularly sensitive population (Bouchaoui et al., 2023; Sun et al., 2023). Moreover, DAergic neurons affected by PD are known to have a particularly high iron content (Friedrich et al., 2021). For an overall evaluation, it is important to note that toxicity by oxidative stress is always dependent on the balance between the generation of reactive oxygen species and the buffering by *e.g.* glutathione and glutathione-dependent enzymatic reactions. Cells differ greatly in their capacity to regenerate glutathione, and neurons are known to be particularly dependent on the uptake of glutathione pre-cursors from extracellular sources. Their own anti-oxidant pool is easily exhausted (Asanuma and Miyazaki, 2021; Ishii et al., 2019; Lotharius et al., 2005). For instance, neurons, including the LUHMES cells used here, recruit a substantial amount of their cysteine from protein turnover. If this is inhibited, glutathione is rapidly depleted (Gutbier et al., 2018b; Suciu et al., 2023). The oxidative stress generated by DA autoxidation may be a further sensitizing factor to explain the selectivity (Sun et al., 2006; Uhl, 2019; Vergo et al., 2007; Xie et al., 2010).

Inhibition of the mitochondrial electron transfer chain (Bose and Beal, 2016; Schapira, 2012) is one of the most-studied pathomechanisms relevant for neurons. On first sight, it may not seem intuitive that DAergic neurons are specifically affected by mitochondrial inhibitors, as most cell types somehow use mitochondria. However, data sets spanning from animal experiments to epidemiological findings support this hypothesis (Schildknecht et al., 2017; Terron et al., 2018), with the most likely explanation being that DAergic neurons depend particularly strongly on mitochondrial function. We considered it necessary to examine this candidate mechanism for aerugine. Using two different experimental approaches, we found that mitochondrial ATP generation is unlikely to be the primary target of the toxicant. Thus, this alternative hypothesis on aerugine toxicity can be excluded.

For many complex human diseases, no laboratory model can cover all aspects. Conversely, it is important for drug discovery to have access to diverse models reflecting different aspects of the pathogenesis. In the field of parkinsonian neurodegeneration, many mitochondrial models have been developed and are in current use. Also, a large panel of genetic models is available. They make use of gene variants found in familial forms of PD (*e.g.,* mutations or altered expression levels of synuclein, parkin, PINK1) (Bose et al., 2022; Chia et al., 2020; Program, 2021) and mimic disturbed proteostasis or impaired mitochondrial function. An aerugine-based model could be an interesting addition that covers complementary aspects. A starting point into this direction could be the exposure of more complex human organoids to the toxin or the probing of species specificity. In parallel, a full toxicological and pharmacological characterization of aerugine and the exploration of related chemical structures are important future research lines. This requires the development of chemical synthesis strategies for related bacterial secondary metabolites and a further characterization of biosynthetic clusters in different microbial communities in humans and the environment.

## Supplementary Material

1

## Data Availability

Data will be made available on request.

## Abbreviations:

BSR: Basal slowing response
DA: Dopamine
dBcAMP: Dibutyryl cyclic adenosine monophosphate
DCM: Dichloromethane
DHAA: Dihydroaeruginoic acid, carboxylic acid of aerugine
dhIQS: Aldehyde of aerugine
DFO: Deferoxamine
DFP: Deferiprone
DMSO: Dimethyl sulfoxide
DoDx: Day x of stem cell differentiation
dx: Day x of differentiation
EA: Ethyl acetate
EC50: Half-maximal effective concentration
FA: Formic acid
FCCP: Carbonyl cyanide-4-(trifluoromethoxy)phenylhydrazone
FCS: Fetal calf serum
FGF-2: Fibroblast growth factor 2
GABA: γ-Aminobutyric acids
GDNF: Recombinant human glial cell derived neurotrophic factor
GFP: Green fluorescent protein
Gpx4: Glutathione peroxidase 4
iPSC: Induced pluripotent stem cells
IQS: Aldehyde of aeruginol
L1: First larval stage
L4: Larval stage 4
LDH: Lactate dehydrogenase
MeOH: Methanol
MPTP: 1-methyl-4-phenyl-1,2,3,6-tetrahydropyridine
NA: Neurite Area
NADH: Nicotinamide-adenine-dinucleotide
NGM: Nematode growth medium
OCR: Oxygen consumption rate
OD: Optical density
PBS: Phosphate buffered saline
PD: Parkinson’s disease
PI: Propidium iodide
PLO: Poly-L-ornithine hydrobromide
QSAR: Quantitative structure-activity relationship
qVD: modified valyl-aspartate, QVD-OPh
R: Resazurin reduction
RIPK: Receptor-interacting serine/threonine-protein kinase
ROS: Reactive oxygen species
SEM: Standard error of the mean
SK4: 3-hydroxy-4(1H)-pyridinone
SOD: Superoxide dismutase
TH: Tyrosine hydroxylase
THF: Tetrahydrofuran
TMRE: Tetramethylrhodamine ethyl ester
VCSA: Virtual cell some area
VCS: Viable cellular structures
