# Supplementary material for: Identification of the bacterial metabolite aerugine as potential trigger of human dopaminergic neurodegeneration
Source: Environ Int. Author manuscript; Available in PMC 2023 Nov 23. (PMC10666548; doi:10.1016/j.envint.2023.108229)
Supplement: 1 [file NIHMS1937915-supplement-1.pdf]

Supplementary information for

Identification of the bacterial metabolite aerugine as potential trigger of human dopaminergic neurodegeneration

Anna-Katharina Ückert[a,1], Sina Rütschlin[b,1], Simon Gutbier[a,1,2], Nathalie Christine Wörz[c,d], Mahfuzur R. Miah[e], Airton C. Martins[e,f], Isa Hauer[a], Anna-Katharina Holzer[a], Birthe Meyburg[a], Ann-Kathrin Mix[g], Christof Hauck[g], Michael Aschner[e,f], Thomas Böttcher[b,c,1,], Marcel Leist[a,1]*

Table of Contents

Fig. S1	P2	Full time course profile of the reverse phase HPLC purification of the active *S. venezuelae* extract
Fig. S2	P3	Structure analysis of aerugine and aeruginol
Fig. S3	P4	Synthesis of aerugine and aeruginol
Fig. S4	P5	Physicochemical properties of aerugine and aeruginol
Fig. S5	P7	Proposed biosynthesis of watasemycin and aerugine
Fig. S6	P9	Thiazoline and thiazole related natural products of different environmental and human-associated bacteria
Fig. S7	P10	Viability endpoints measured on d6 LUHMES treated with aerugine
Fig. S8	P11	Images of d6 LUHMES cells after aerugine exposure for 8 h
Fig. S9	P12	No interaction of aerugine with the mitochondrial respiratory chain
Fig. S10	P14	Neurotoxicity of aerugine analogues
Fig. S11	P15	Toxicity of aerugine on LUHMES neurons in various differentiation stages
Fig. S12	P16	DFP prevents aerugine-induced cell death in d6 LUHMES, but not loss of neurites
Fig. S13	P17	Degeneration scores of GABAergic neurons in *C. elegans*
Spectral data 1	P18	Spectral data for structure elucidation: 1H NMR, 13C NMR, COSY spectra, HSQC spectra, HMBC spectra of aerugine and aeruginol
	P32	Supplementary references

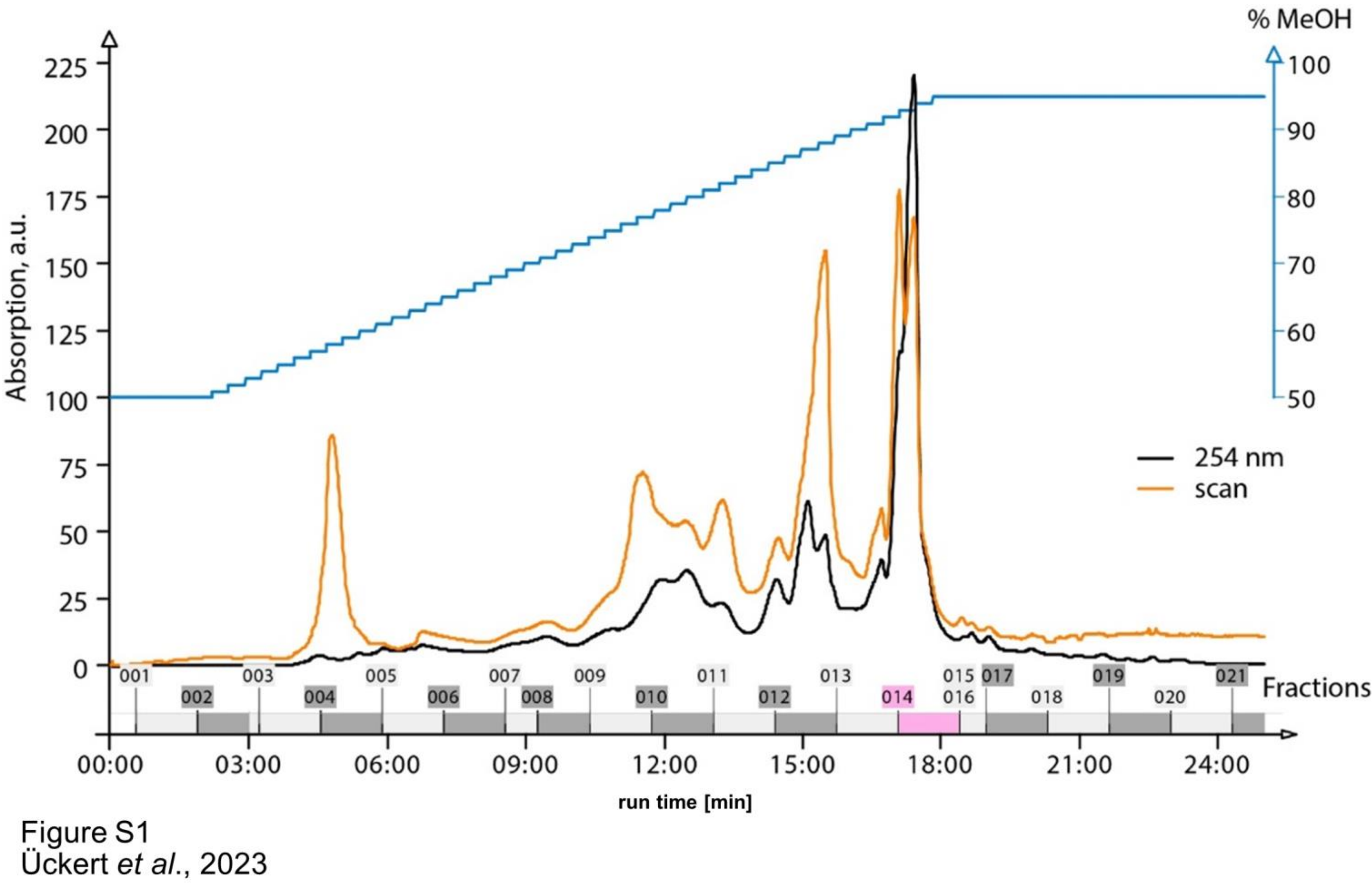

Supplementary Figure S1: Full time course profile of the reverse phase HPLC purification of the active S. venezuelae extract

The MeOH gradient is indicated in blue. The multi wavelength scan is given in orange. Fractions (indicated at the bottom) were obtained by automatic peak detection. The fraction used in the further purification is indicated in pink. A part of this spectrum is shown with the corresponding viability data in figure 1.

A

^{1}H NMR and ^{13}C NMR data for extracted aerugine and aeruginol

	Aerugine		Aeruginol	
Structure	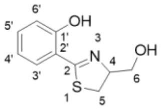		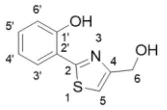	
Position	δ_C	δ_H (J Hz)	δ_C	δ_H (J Hz)
2	171.05		164.41	
4	77.83	4.81 m	156.55	
5a	32.58	3.31 m	114.54	7.44 s
5b		3.49 dd (10.8, 8.9)		
6	62.32	3.63 m	59.62	4.62 s
-OH		5.11 t (5.6)		5.38 s
1‘	158.41		155.79	
2‘	115.82		119.05	
3‘	130.32	7.41 m	127.24	7.98 m
4‘	116.70	6.97 dd (8.9, 1.2)	116.84	7.01 d (8.2)
5‘	133.21	7.41 m	130.95	7.28 t (7.6)
6‘	119.10	6.94 td (7.6, 1.2)	118.62	6.90 t (7.4)
Ph-OH		12.65		11.65 s

B

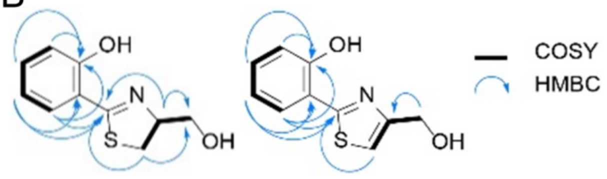

δ_C = ^{13}C NMR chemical shift
δ_H (J Hz) = ^{1}H NMR chemical shift

Figure S2
Ückert *et al.*, 2023

Supplementary Figure S2: Structure analysis of aerugine and aeruginol

^{1}H NMR (400 MHz), ^{13}C NMR, COSY, HSQC and HMBC spectra were recorded in DMSO-d_6. **A** Summary of ^{1}H NMR and ^{13}C NMR data for extracted aerugine and aeruginol. **B** Interpretation of COSY and HMBC data of aerugine and aeruginol. Details are found in the “raw data 1” section.

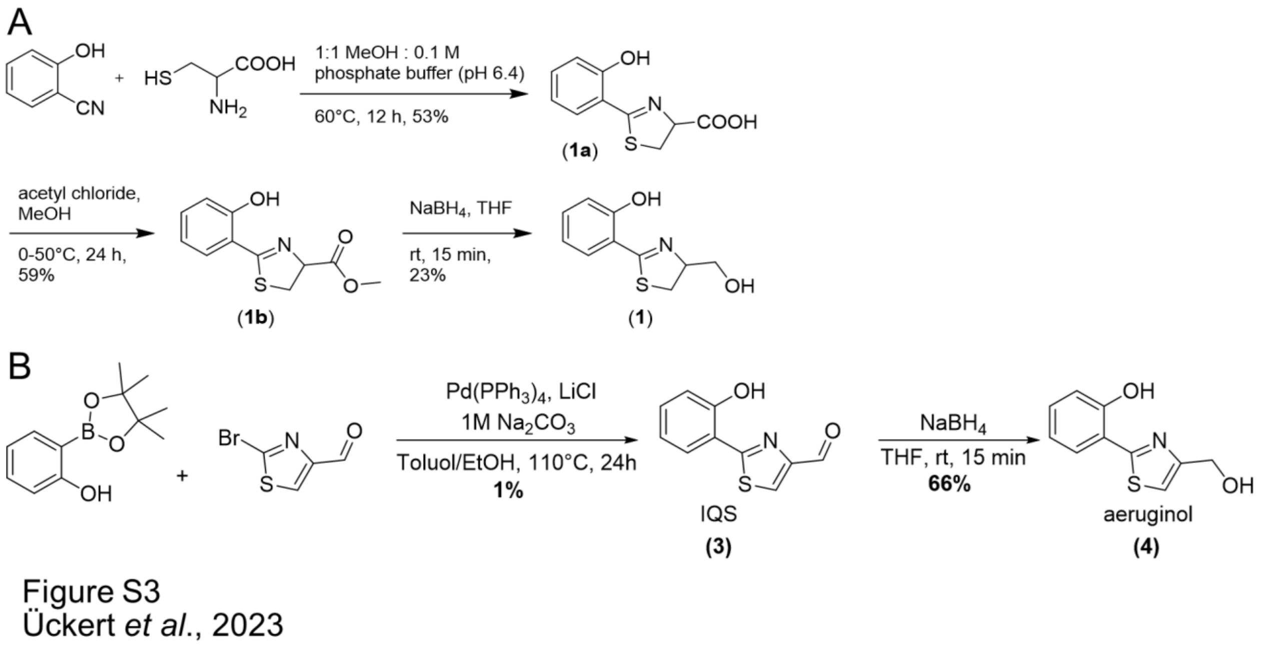

Supplementary Figure S3: Synthesis of aerugine and aeruginol

A Synthesis of aerugine (**1**) by condensation of ʟ-cysteine and 2-hydroxybenzonitrile to (**1a**) following methyl ester formation (**1b**) and reduction to the alcohol. **B** Synthesis of aeruginol via the reduction of IQS.

	Aerugine	Aeruginol
CAS.№		154037-50-0
Smile structure	C1C(N=C(S1)C2=CC=CC=C2O)CO	C1=CC=C(C(=C1)C2=NC(=CS2)CO)O
Structure and pKA values	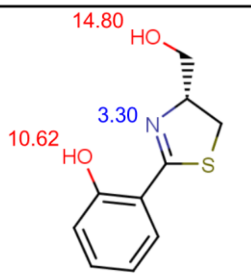	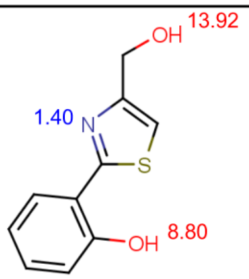
Structural formula	$C_{10}H_{11}NO_2S$	$C_{10}H_9NO_2S$
Molecular weight (g/mol)	209.27	207.25
logP (KOWWIN, 1.69 ver)	2.32	1.36
Boiling point (stein & brown method)	366.9	385.8
Melting point (mean)	140.72	153.07
Henry's law constant @25°C ($Pa\text{-}m^3/mol$) (Bond method)	9.43×10^{-10}	1.25×10^{-10}
Log Kaw (air : water) (Henry win est)	-12.4	-13.3
Log Koa (octanol : air) @25°C (KOAWIN, v 1.10)	14.7	14.7
Water solubility @25°C (mM) (from LogP value)	7.4	51
Effective membrane permeability (cm/s) [-$LogP_{eff}$]	$3.0 \cdot 10^{-4}$ [3.52]	$2.6 \cdot 10^{-4}$ [3.58]
Membrane affinity (logK(neutral-phospholipids : water))	2.4	1.6
Blood brain barrier passage	yes	yes

Figure S4
Ückert *et al.*, 2023

Supplementary Figure S4: Physicochemical properties of aerugine and aeruginol

The table summarizes key compound features of relevance for toxicological prediction models. Below some explanations and reference points are given. The effective membrane permeability (P_{eff}) refers to the rate at which compounds are able to cross biological membranes. This parameter has been used for assessing intestinal absorption. The model used here to predict the permeability of compounds was suggested by Winiwarter *et al.* (Winiwarter et al., 1998). Membrane permeability is predicted from the compound's $LogD_{5.5}$ (LogP at pH 5.5), the topological surface area and hydrogen bond donor counts. A low P_{eff} value is associated with poor membrane permeability. The model parameterization is as follows: permeable molecules (reaching the cytosol independent of transporters) (Cooper, 2000), such as benzene (hydrophobic), ethanol (small polar but uncharged) and nicotine have predicted values (in -$LogP_{eff}$) of 2.5, 3.37 and 3.44 respectively; impermeable molecules (requiring a transporter to enter the cytosol) such as glucose, glutamate and dopamine have predicted values of 5.81, 5.16 and 4.77 respectively.

The blood brain barrier passage was predicted as suggested by Liu *et al.* (Liu et al., 2014) and used and explained in Marques *et al.* and de Souza-Fagundes *et al.* (de Souza-Fagundes et al., 2018; Marques et al., 2020). This

model assigns scores to compounds. The scores do not predict the extent of permeation, but categorize compounds as “not passing the BBB” or as being permeable. At physiological pH, 7.4, prediction of the major microspecies using ChemAxon version 17.1.23 shows that both aerugine and aeruginol exist as non-ionised compounds. They are predicted to pass the blood brain barrier (=yes).

The term “membrane affinity” describes the distribution of a compound from an aqueous solution to phospholipid membranes. The values for membrane affinity at 37°C are predicted using a simple regression model based on the compound logP (Endo et al., 2011; Pearce et al., 2017). The predicted value is a phosphotidylcholine: water partition coefficient at pH 7.4, with the general rule being that the higher the logP the higher the membrane affinity. The affinity values predicted for aerugine and aeruginol are in a similar range as the predicted values of benzene (2.3) and ethanol (1.4). Rotenone has an even higher membrane affinity (4.0). Dopamine, glutamate and glucose have a low membrane affinity, which is reflected in their predicted values of -0.5, -2.9 and -2.5 respectively.

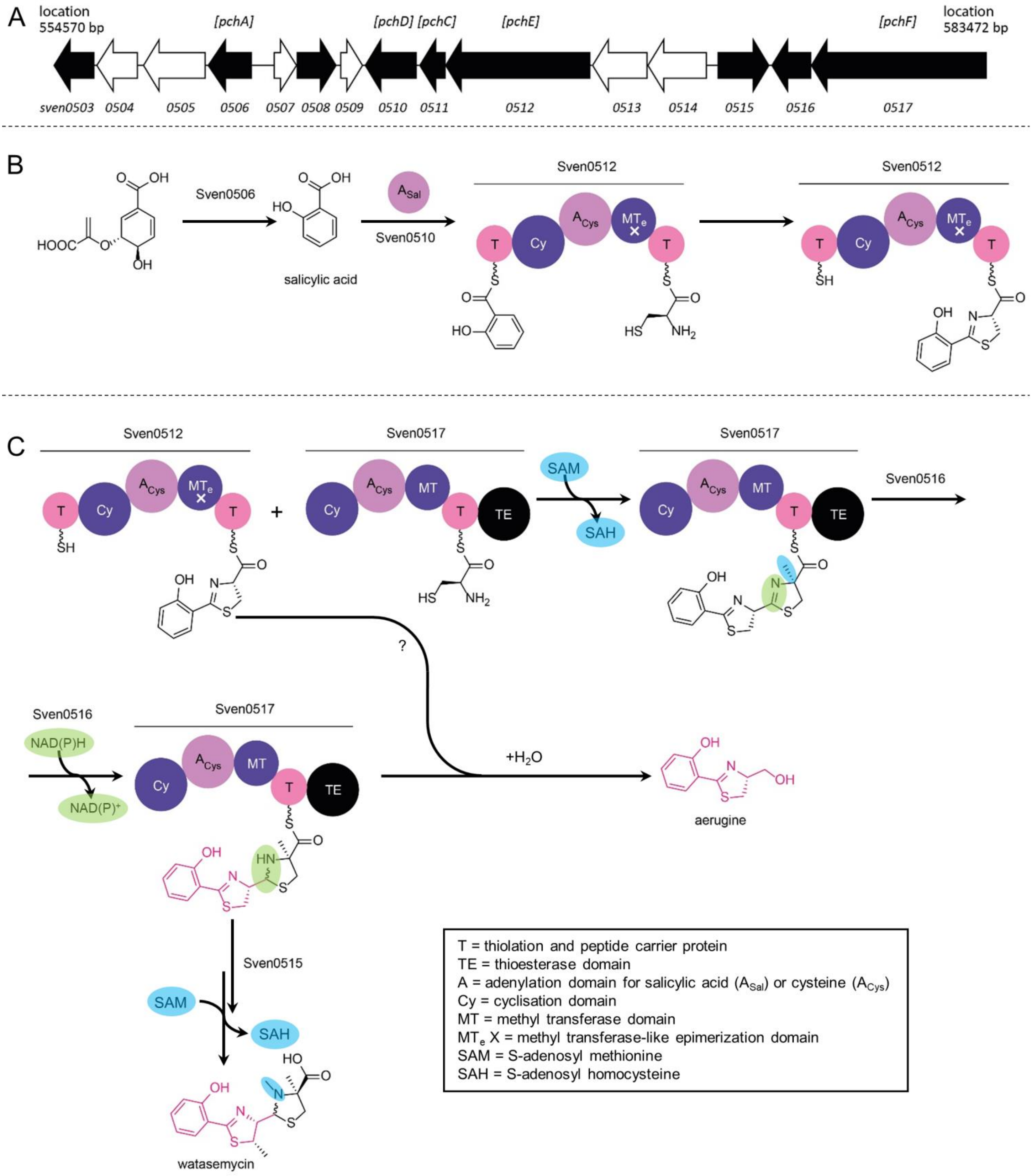

Figure S5
Ückert *et al.*, 2023

Supplementary Figure S5: Proposed biosynthesis of watasemycin and aerugine.

A Biosynthetic gene cluster of watasemycin of *Streptomyces venezuelae* ATCC 10712 investigated via antiSMASH. Homologs of the pyochelin gene cluster of *Pseudomonas aeruginosa* are indicated in brackets; black arrows: genes encoding biosynthetic enzymes, white arrows: genes with transport and regulatory functions. **B** Proposed mechanism: the PchA homolog Sven0506 is involved in production of salicylic acid, which is loaded with help of the lone adenylation domain Sven0510 on the nonribosomal peptide synthetase (NRPS) module Sven0512. The MT_e domain of Sven0512 is nonfunctional. Condensation with cysteine gives the thiazoline ring. **C** This is transferred and condensated with a second cysteine and methylated via module Sven0517. The reductase Sven0516 generates a thiazolidine ring and further methylation leads to watasemycin. Aerugine is presumably produced by hydrolysis of the thiazolidine ring. Gene cluster and mechanism adapted and modified from Inahashi et al. (Inahashi et al., 2017)

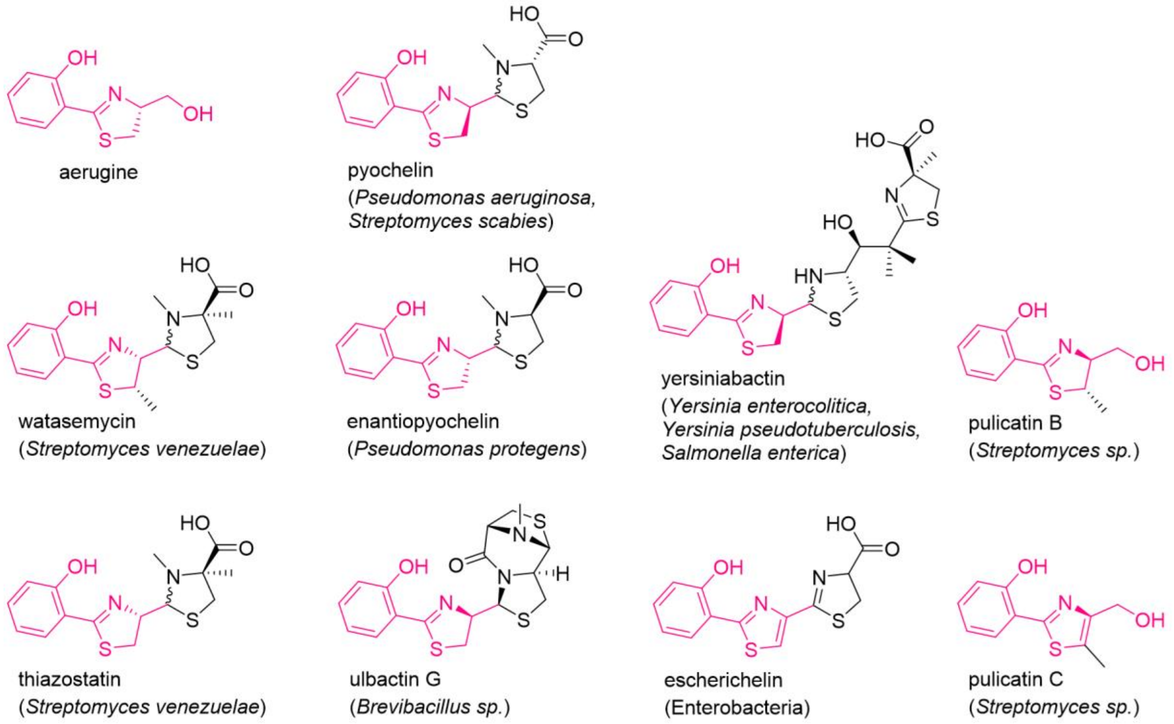

Figure S6
Ückert *et al.*, 2023

Supplementary Figure S6: Thiazoline and thiazole related natural products of different environmental and human-associated bacteria

Thiazoline/thiazole-containing compounds were extracted from literature. Their highly homologous biosynthetic pathways suggest that aerugine or aeruginol could be produced as potential "shunt" products. Structures were described in the following references: Watasemycin, (Sasaki et al., 2002) thiazostatin, (Shindo et al., 1989) ulbactin G, (Igarashi et al., 2016) yersiniabactin, (Drechsel et al., 1995) pyochelin, (Seipke et al., 2011) enantiopyochelin, (Youard et al., 2007) and pulicatins B and C. (Lin et al., 2010)

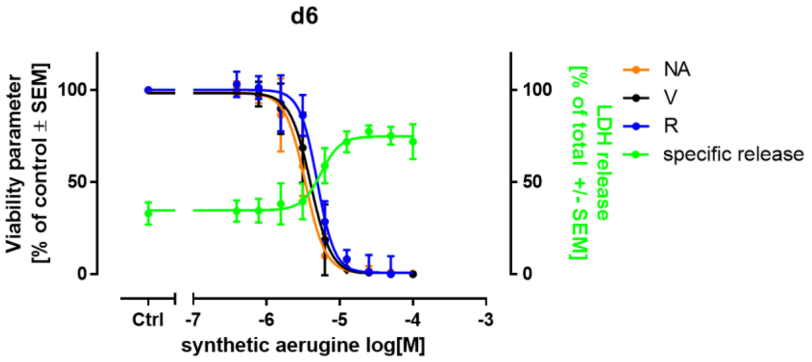

Figure S7
Ückert *et al*., 2023

Supplementary Figure S7: Viability endpoints measured on d6 LUHMES treated with aerugine

Cultures of differentiated LUHMES neurons were exposed on d6 to serial dilutions of aerugine for 24 h. The cell viability (V), neurite area (NA) were assessed 24 h later by calcein-AM & H-33342 staining and high content imaging. Resazurin reduction and LDH release were measured subsequently. Data are presented as means ± SEM of at least three biological replicates

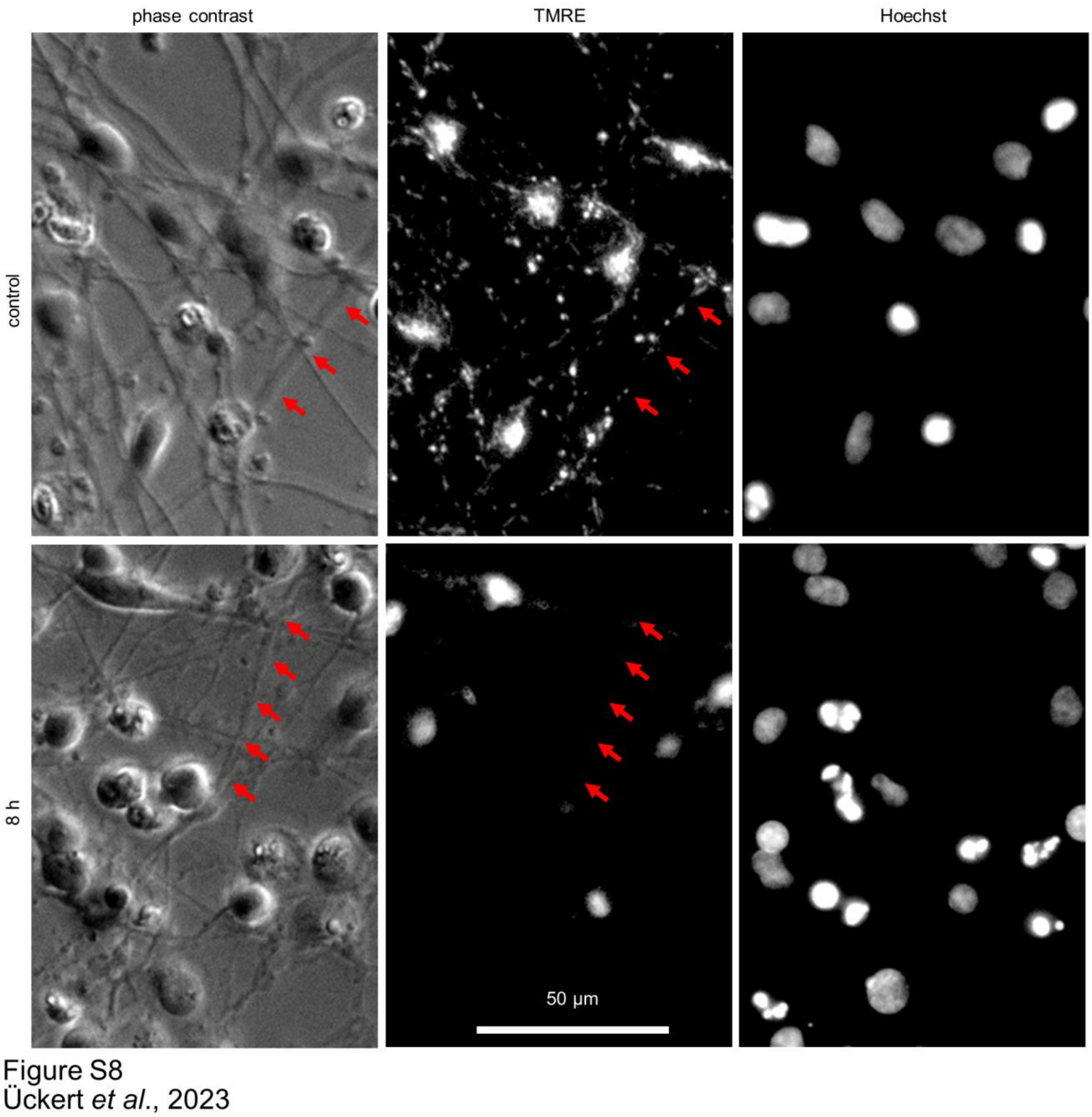

Supplementary Figure S8: Images of d6 LUHMES cells after aerugine exposure for 8 h

LUHMES neurons (d6) were treated with 10 µM aerugine. After the indicated incubation times, they were stained with H-33342 & the mitochondrial membrane potential indicator tetramethylrhodamin-methylester (TMRE). Representative images after exposure to aerugine are shown. Cells reacted similarly to purified aerugine. Scale bar represents 50 µm. Arrows indicate an intact neurite without functional mitochondria. Images are enlargements of micrographs presented in Fig. 2.

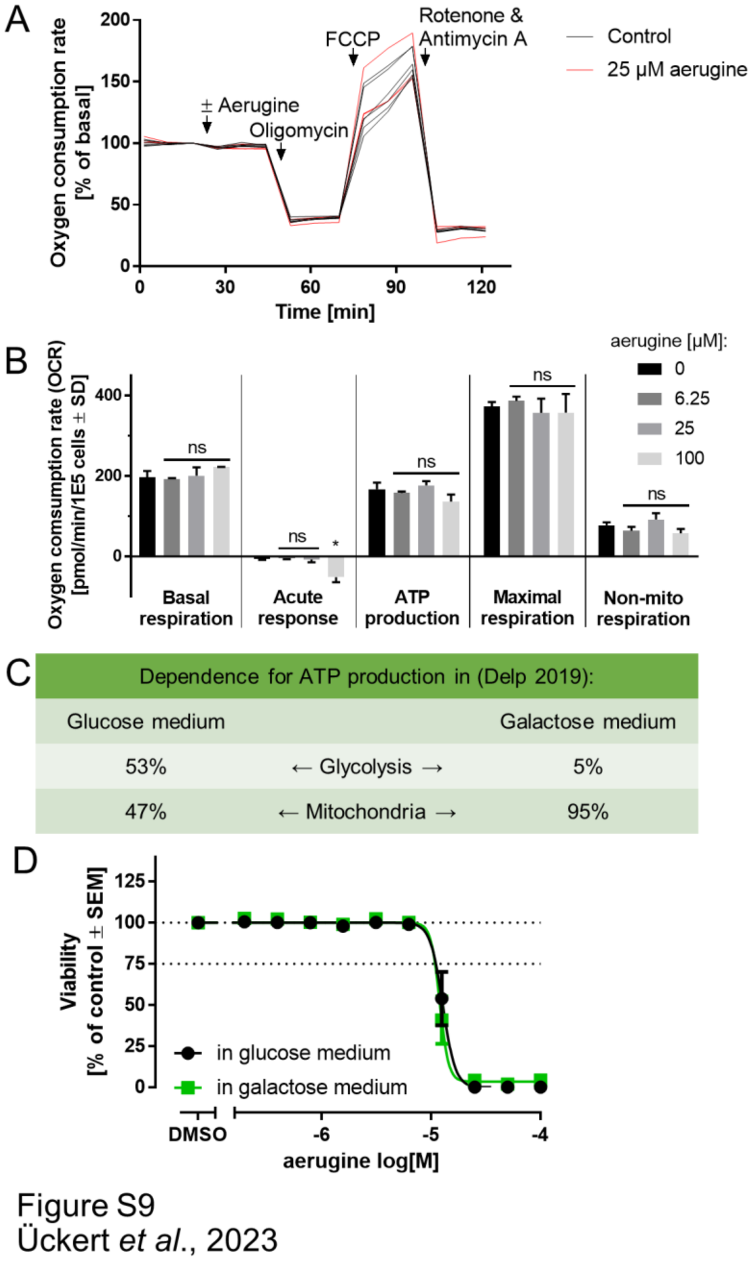

Supplementary Figure S9: No interaction of aerugine with the mitochondrial respiratory chain

A The effect of aerugine on respiratory function was analysed in d3 LUHMES cells. The oxygen consumption rate of the individual technical replicates is shown. The oxygen consumption rate is normalised to the last measurement before injection. The injections of aerugine, control and the subsequent assay tool compounds are indicated (FCCP: 1.5 µM; oligomycin: 1 µM; rotenone + antimycin A: 0.5 µM +0.5 µM). **B** Several parameters were derived from the oxygen consumption rate. The data is presented as means ± SD of four replicates (obtained from one cell batch). Statistical analysis was performed using a one-way ANOVA with Dunnett's multiple comparisons test. * = $p < 0.0001$. **C** Delp *et al.* (2019) determined the dependence of LUHMES cells on glycolysis and their mitochondria for ATP production in two different media conditions. Since the cells become more dependent on their mitochondria when supplied with galactose instead of glucose, mitochondrial toxicants are 100-1000fold more cytotoxic under these conditions. (Delp et al., 2019) Under both conditions, the overall cellular ATP content was similar, and the numbers indicate the contributions of glycolysis vs

mitochondrial metabolism in producing ATP in the two different media. **D** Aerugine toxicity was assessed in d3 LUHMES cells cultured in glucose and in galactose medium. Cell viability was assessed by calcein-AM & H-33342 staining and automated fluorescence microscopy. Double positive cells were counted as viable, H-33342 mono-positive cells were counted as dead.

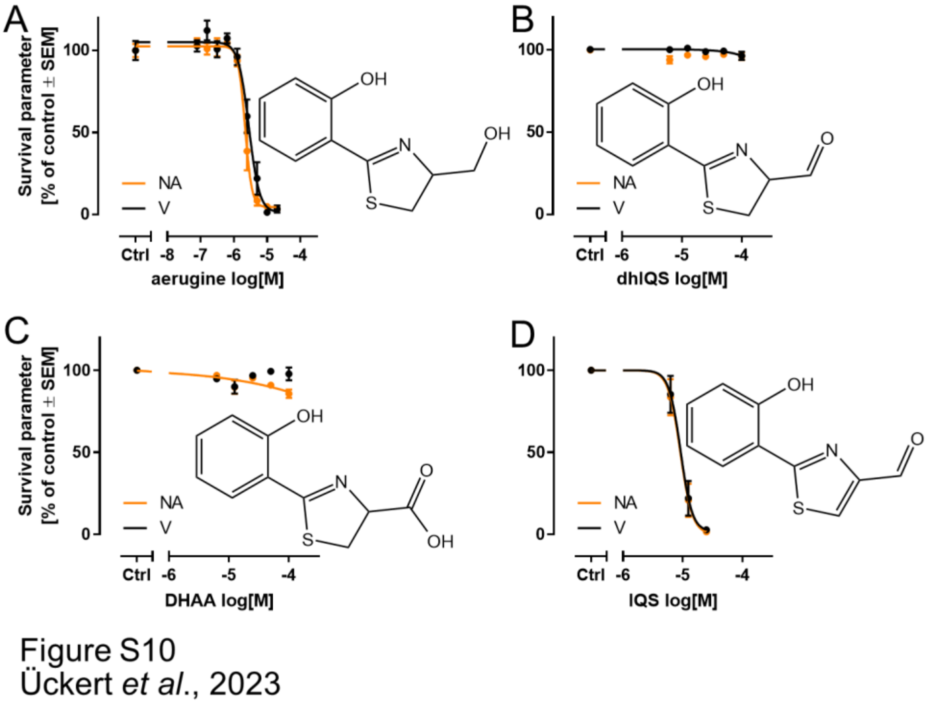

Supplementary Figure S10: Neurotoxicity of aerugine analogues

Cultures of differentiated LUHMES neurons were exposed on d6 to serial dilutions of dihydro-aeruginoic acid (DHAA), dihydro-aeruginealdehyde (dhIQS), aeruginealdehyde (IQS) and aerugine (structures are given). The cell viability (V) and neurite area (NA) were assessed 24 h later by calcein-AM & H-33342 staining and high content imaging. Data are presented as means ± SEM of at least three biological replicates. All compounds were measured in the same three cell lots.

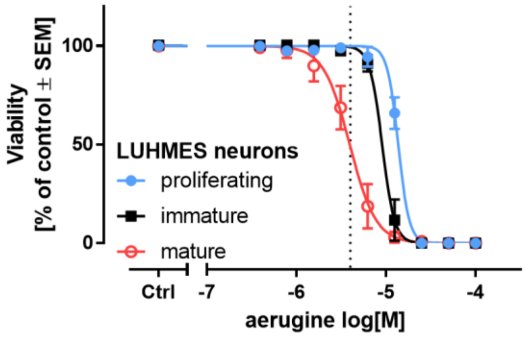

LUHMES neurons	EC50 ± SEM
Mature	4.1 ± 0.8 µM
Immature	9.3 ± 1.1 µM
Proliferating	13.1 ± 0.2 µM

Figure S11
Ückert *et al.*, 2023

Supplementary Figure S11: Toxicity of aerugine on LUHMES neurons in various differentiation stages

Cultures of LUHMES neurons were used on d0 (proliferating, blue), d2 (immature, black) or d6 (mature, red) for cytotoxicity testing. Cells were exposed to 9 serial dilutions of synthetic aerugine. The cell viability was assessed 24 h later by calcein-AM & H-33342 staining and high content imaging. Data are presented as means ± SEM. The average EC50s and their respective SEMs are given in the table below. The sigmoidal fits of the individual experiments were shown in figure 3.

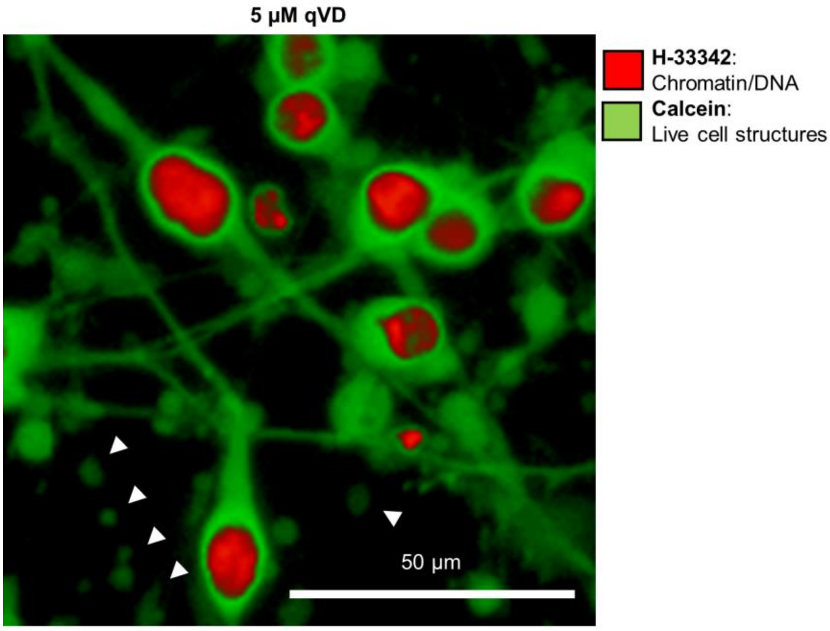

Figure S12
Ückert *et al.*, 2023

Supplementary Figure S12: DFP prevents aerugine-induced cell death in d6 LUHMES, but not loss of neurites

LUHMES cultures (d6) were used under standard conditions to assess the cytotoxicity of aerugine (10 µM) in the presence of 5 µM qVD (modified valyl-aspartate). qVD prevented cell death, but neurite integrity was still lost. Arrow heads indicate a disintegrating neurite (chains of calcein-positive blebs). The image is an enlargement of a micrographs presented in Fig. 4.

.

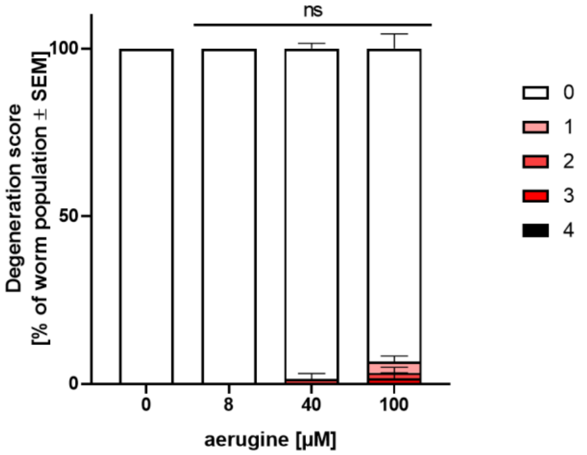

Figure S13
Ückert *et al.*, 2023

Supplementary Figure S13: Degeneration scores of GABAergic neurons in C. elegans

L4 larval stage worms of Punc-25 *C. elegans* (containing fluorescently labelled γ-aminobutyric acid neurons) were treated for 2 days with aerugine. The labelled neurons of 20-30 worms per condition were analyzed and scored for specific neurodegeneration. Each worm was assigned a degeneration score (0 for wild type and 1-4 for increasing severity). In degeneration score 1, dendrites are thinning. When the dendrites turn to blebs (irregular protrusions of the dendrites), degeneration score 2 is reached. If more than 4 blebs occur, degeneration score 3 is indicated. Degenerating cell bodies occur in degeneration stage 4. These scores were assessed to quantify the GABAergic neurodegeneration caused be aerugine *in vivo*.

Spectral data 1: Spectral data of structure elucidation: ^{1}H NMR, ^{13}C NMR, COSY spectra, HSQC spectra, HMBC spectra of aerugine and aeruginol

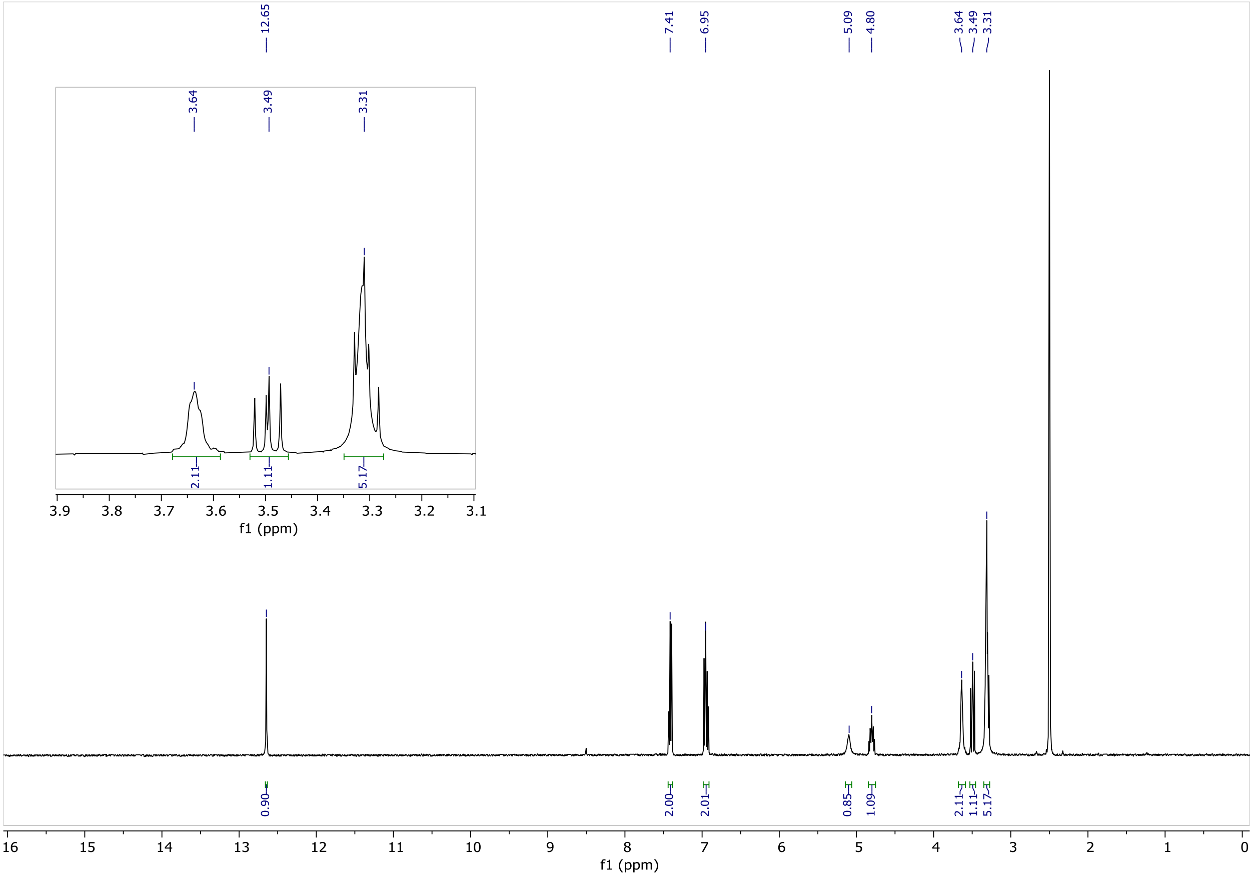

^{1}H NMR in d_6-DMSO (400 MHz) of extracted aerugine.

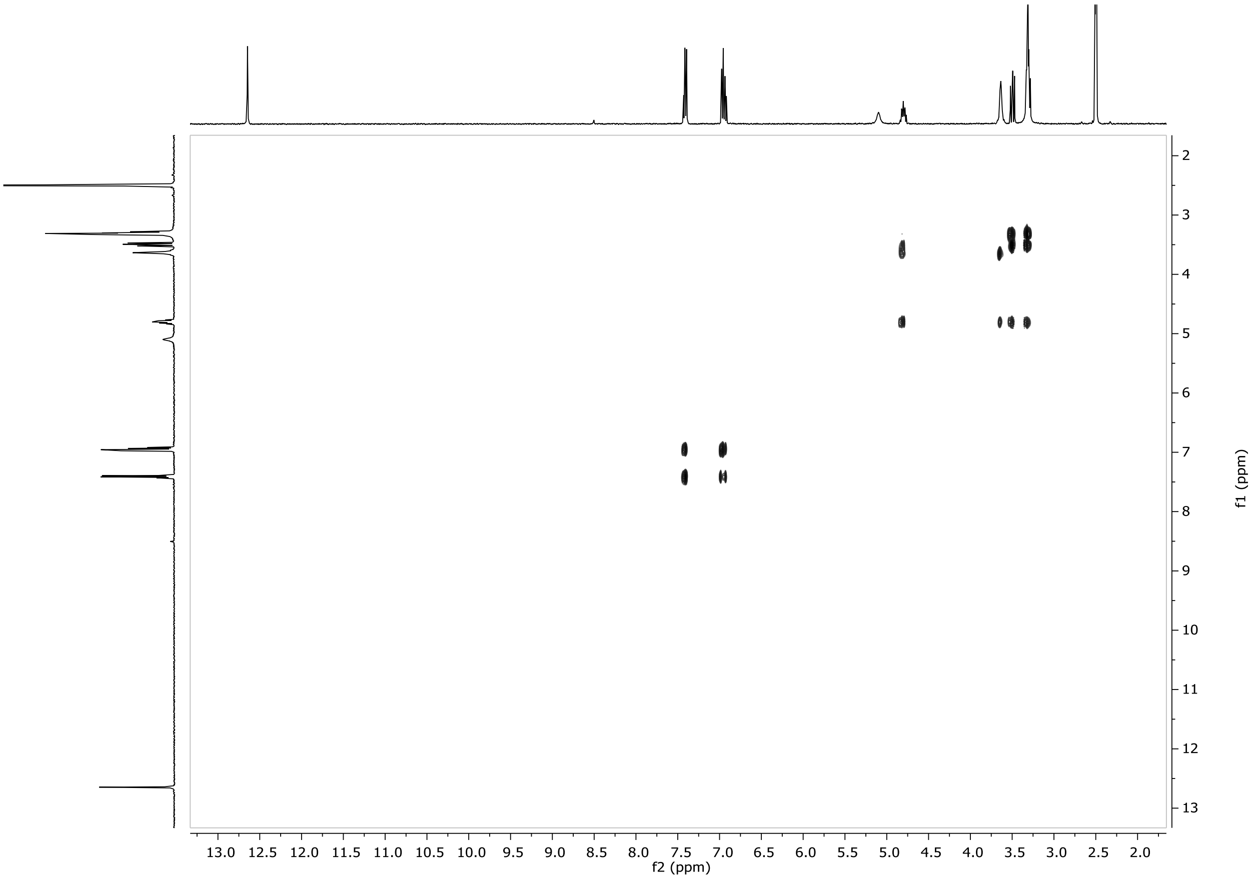

COSY spectrum in d_6-DMSO of extracted aerugine.

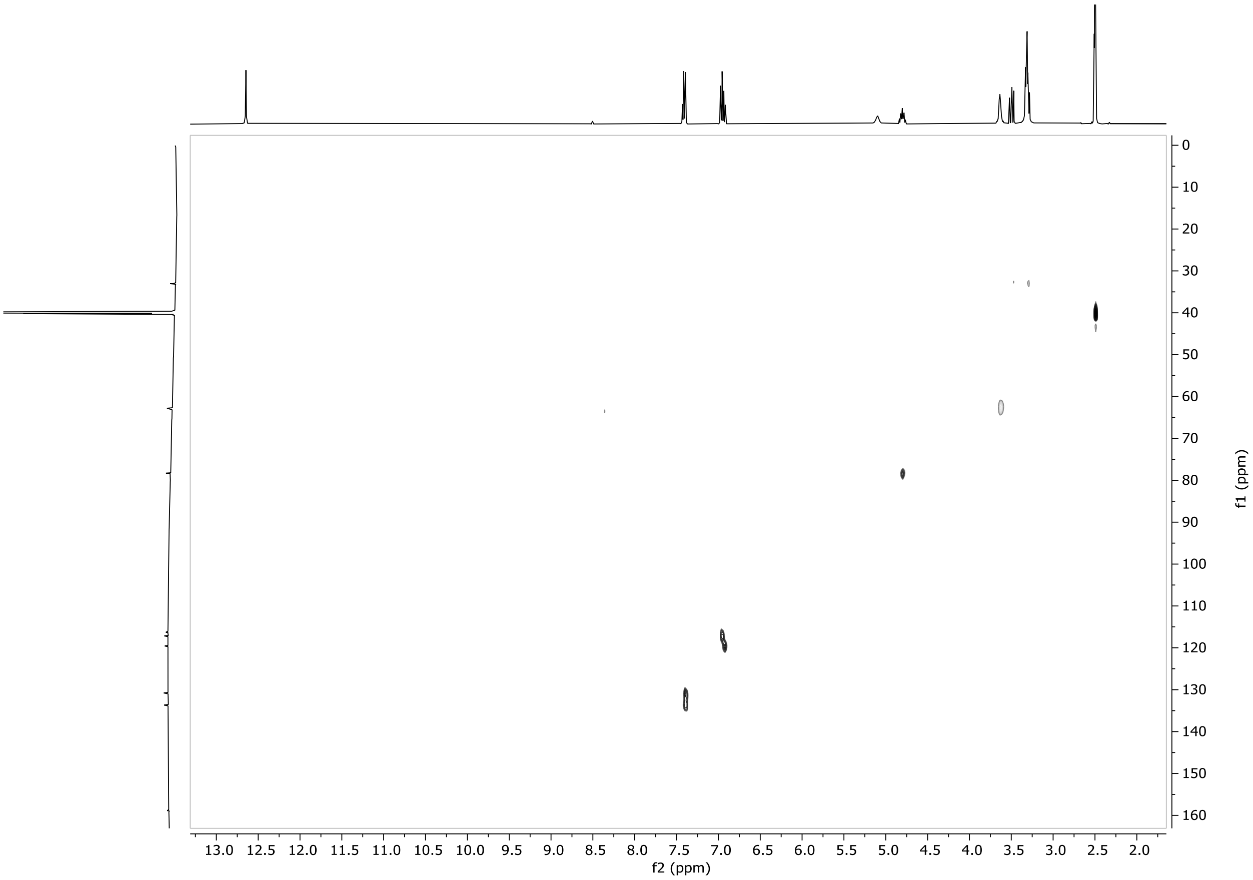

HSQC spectrum in d_6-DMSO of extracted aerugine.

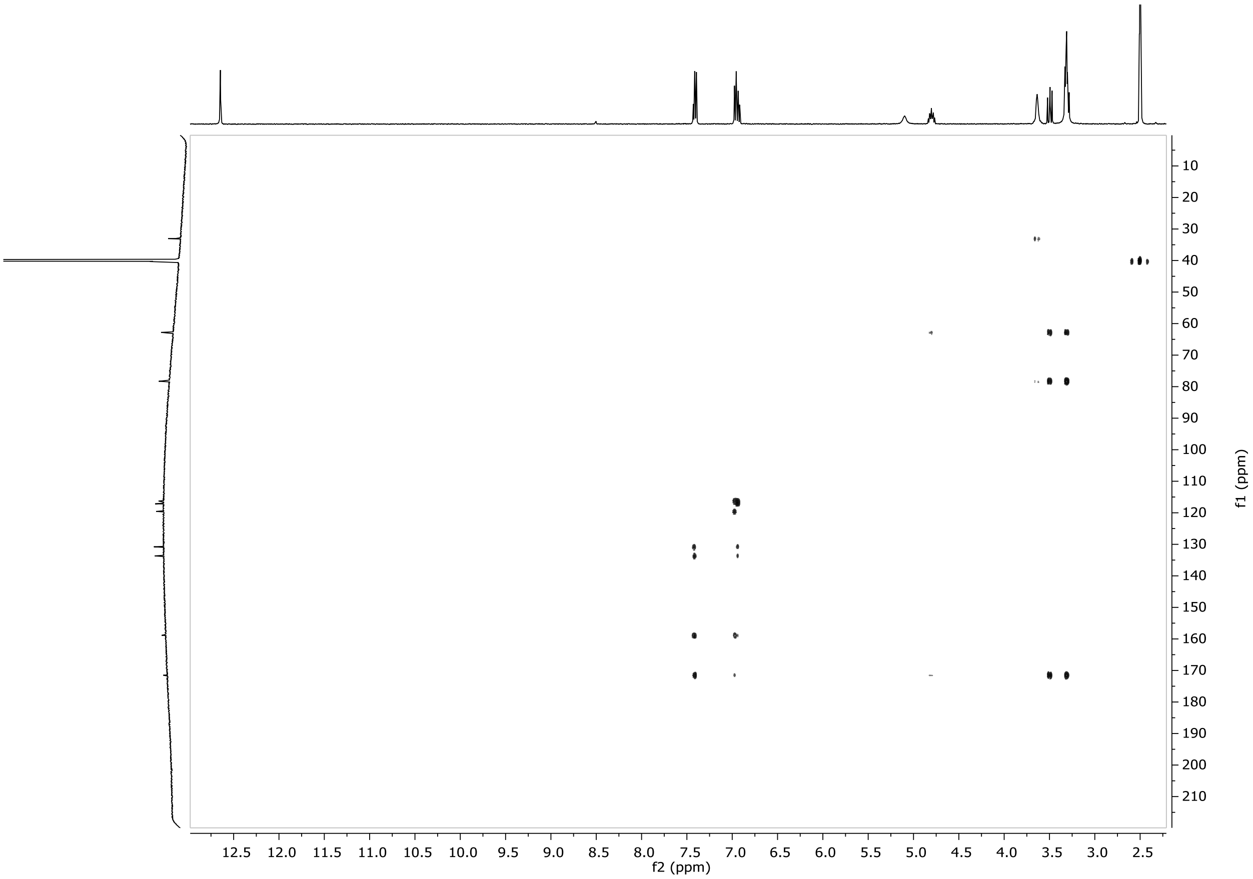

HMBC spectrum in d_6-DMSO of extracted aerugine.

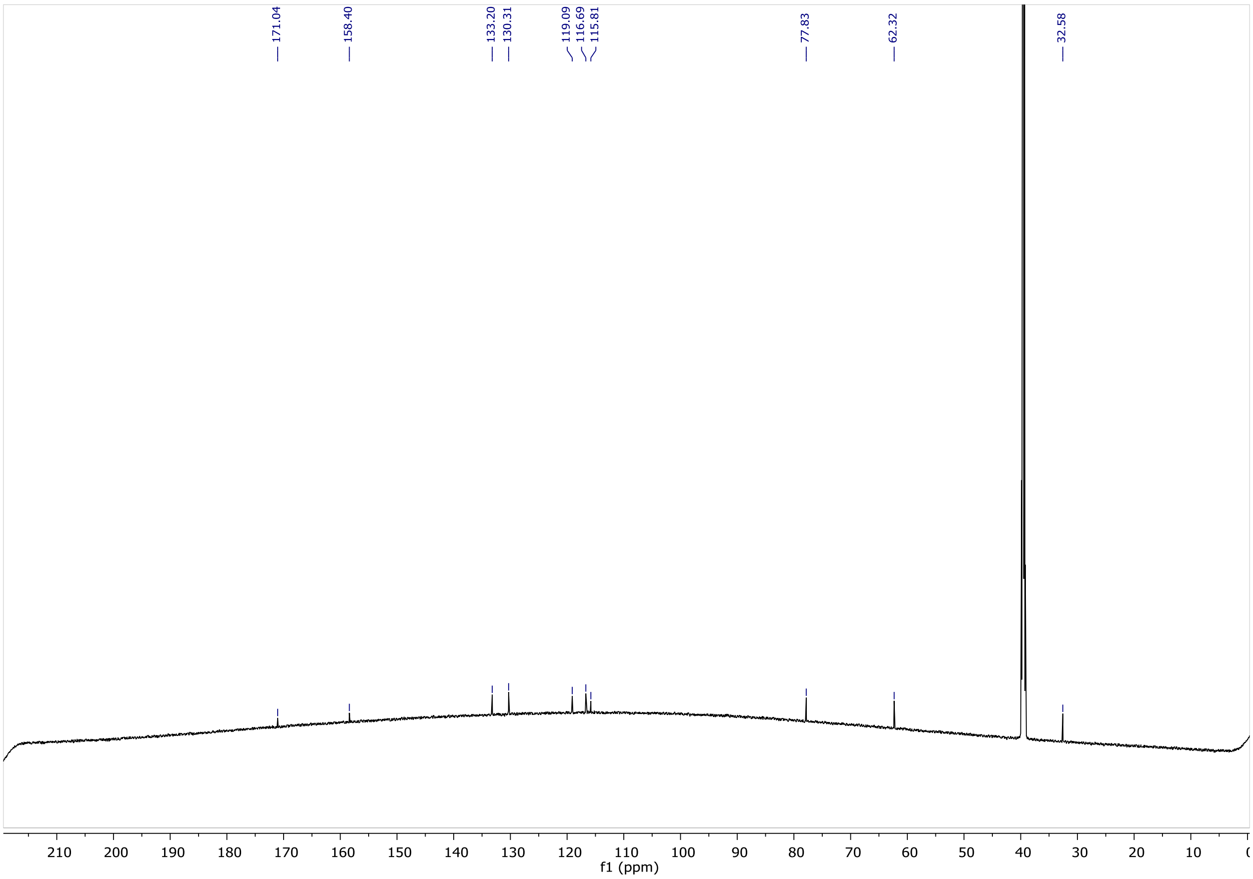

^{13}C NMR in d_6-DMSO of extracted aerugine.

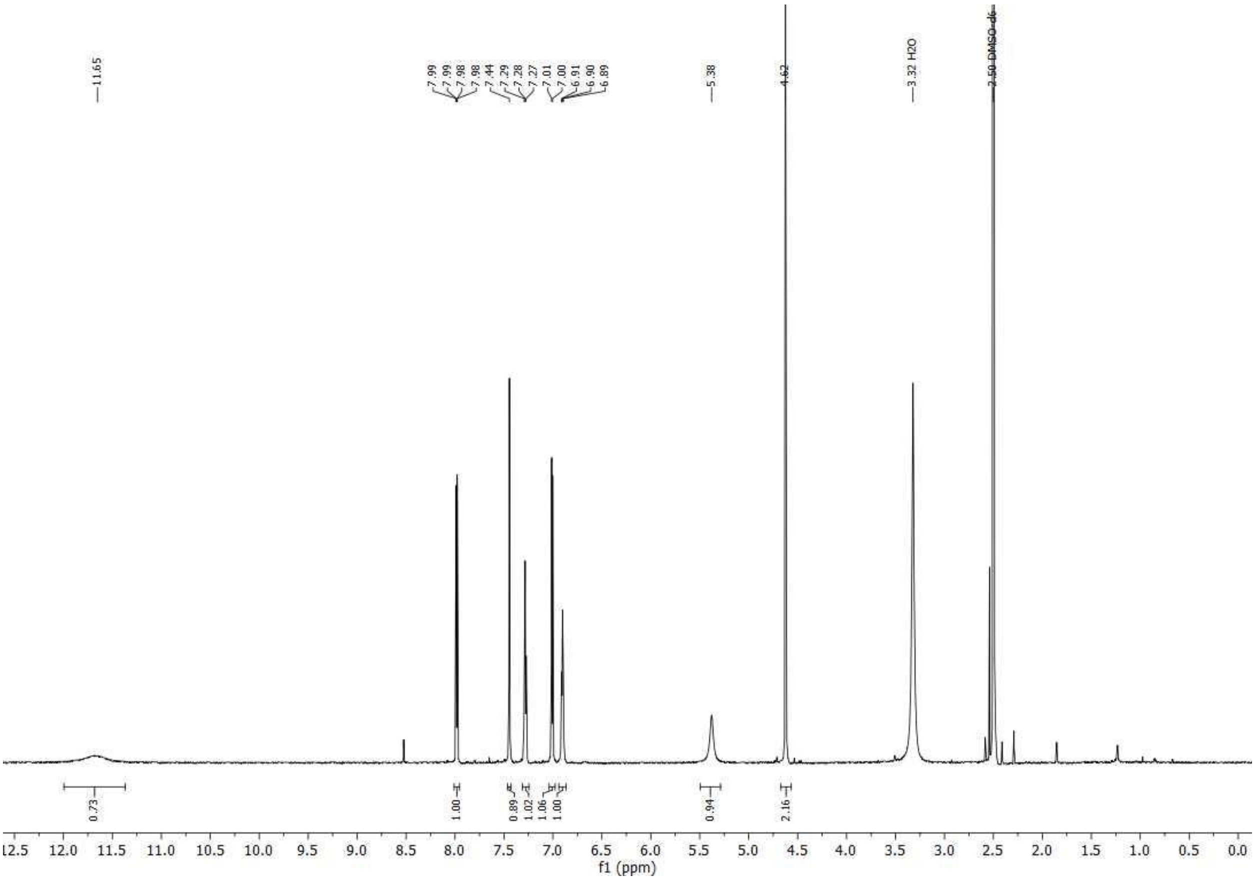

^{1}H NMR in d_6-DMSO 800 MHz of extracted aeruginol.

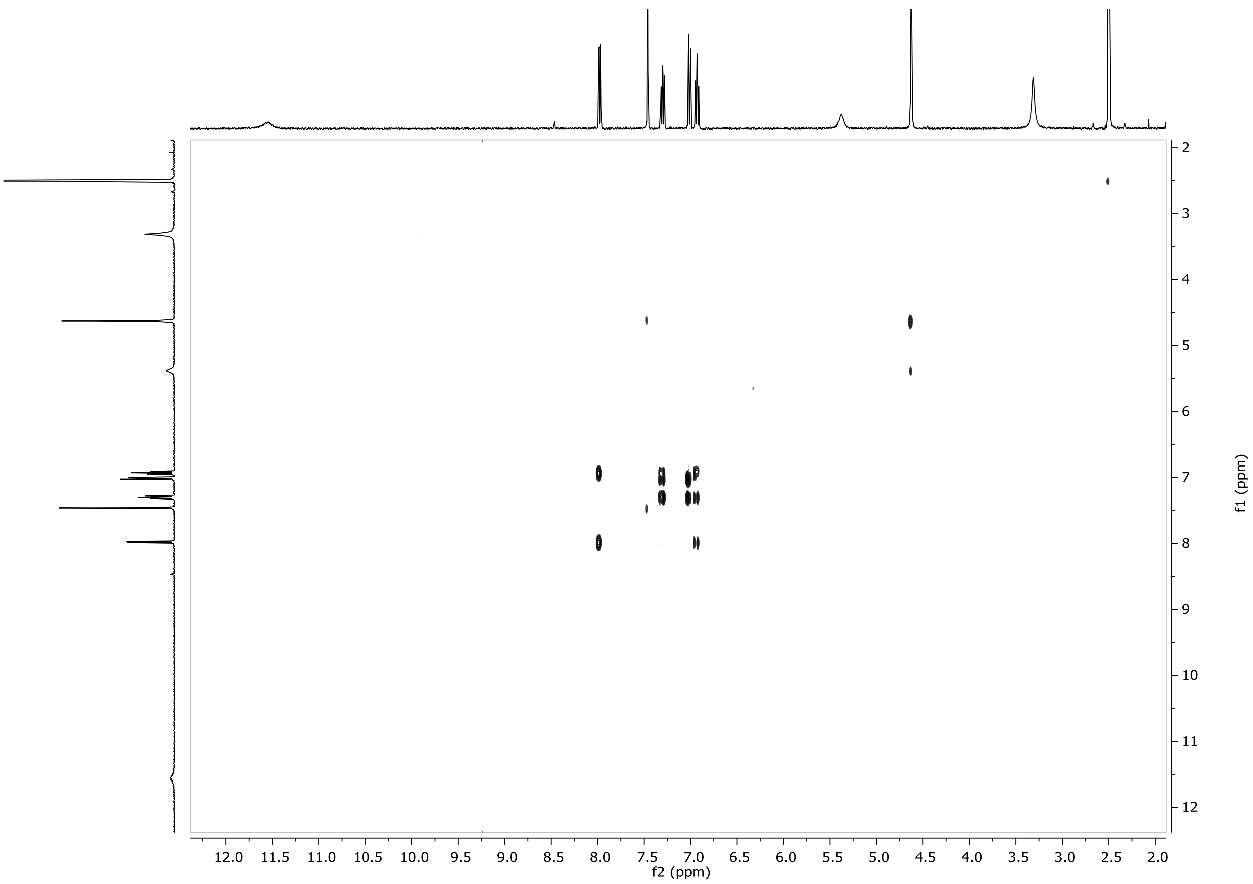

COSY spectrum in d_6-DMSO of extracted aeruginol.

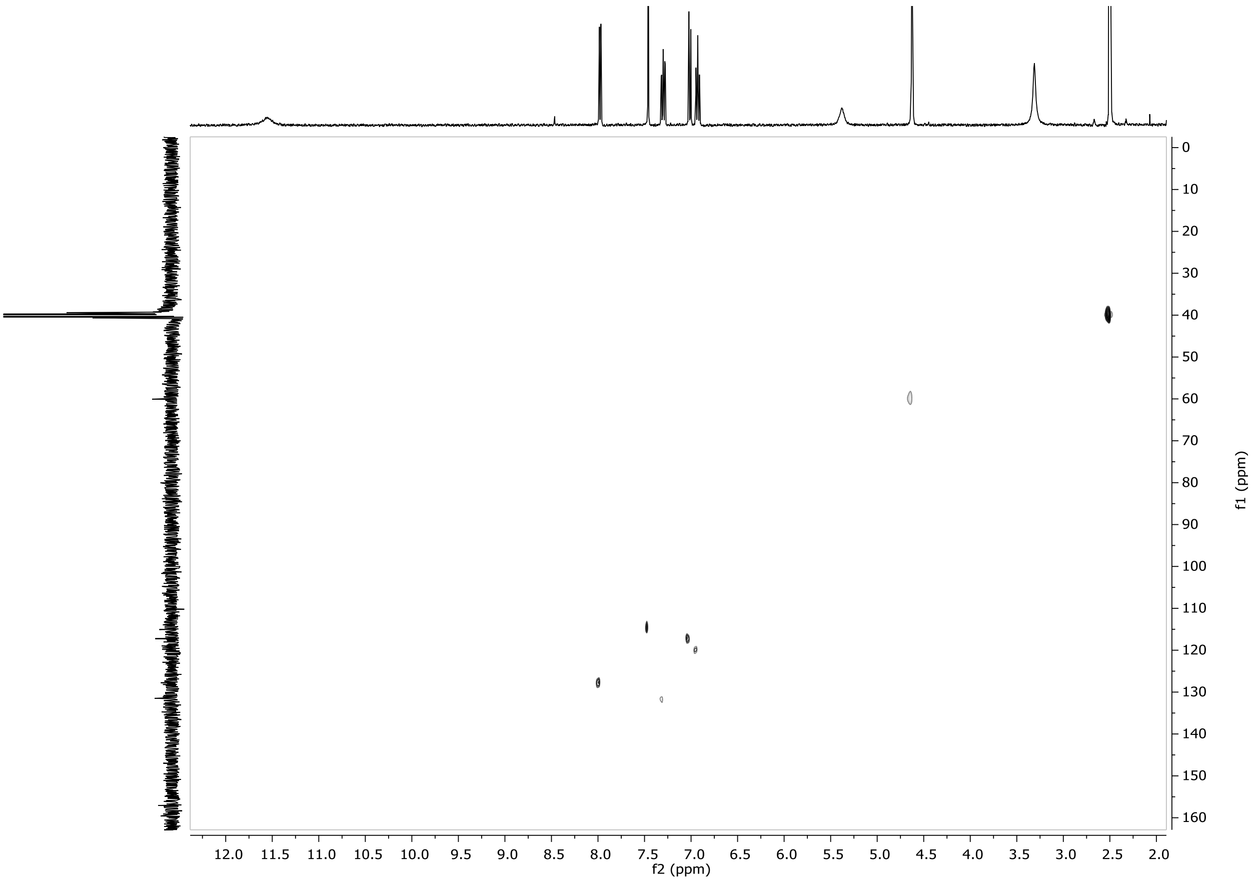

HSQC spectrum in d_6-DMSO of extracted aeruginol.

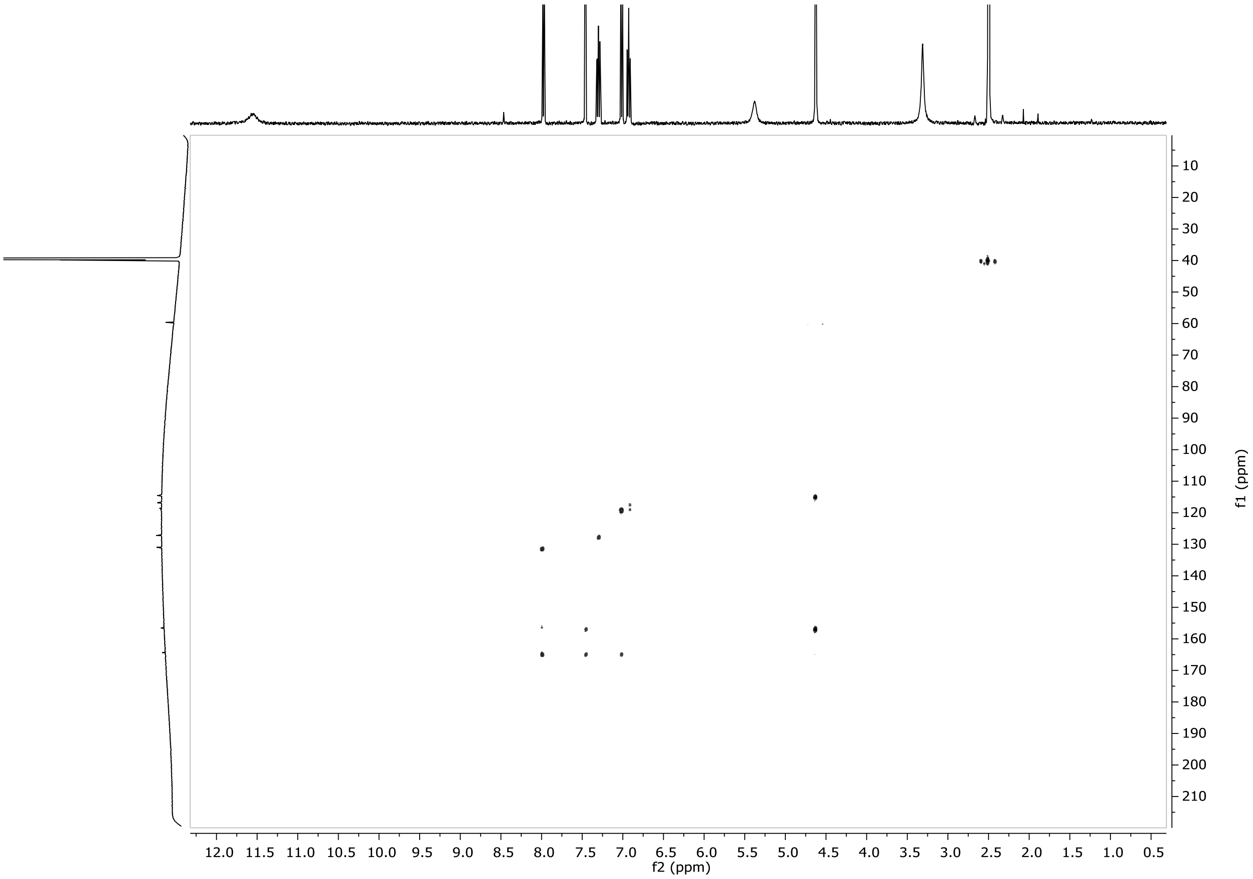

HMBC spectrum in d_6-DMSO of extracted aeruginol.

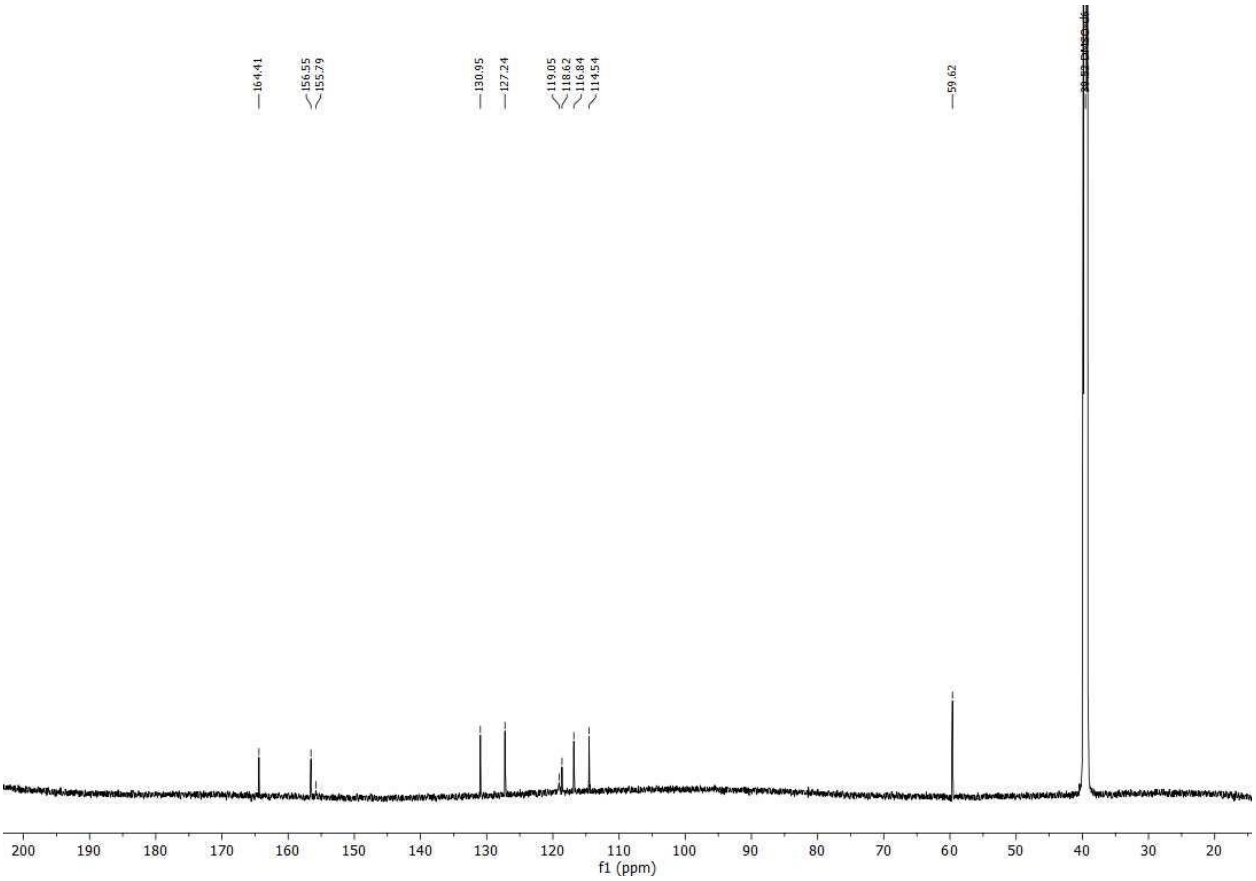

^{13}C NMR in d_6-DMSO of extracted aeruginol.

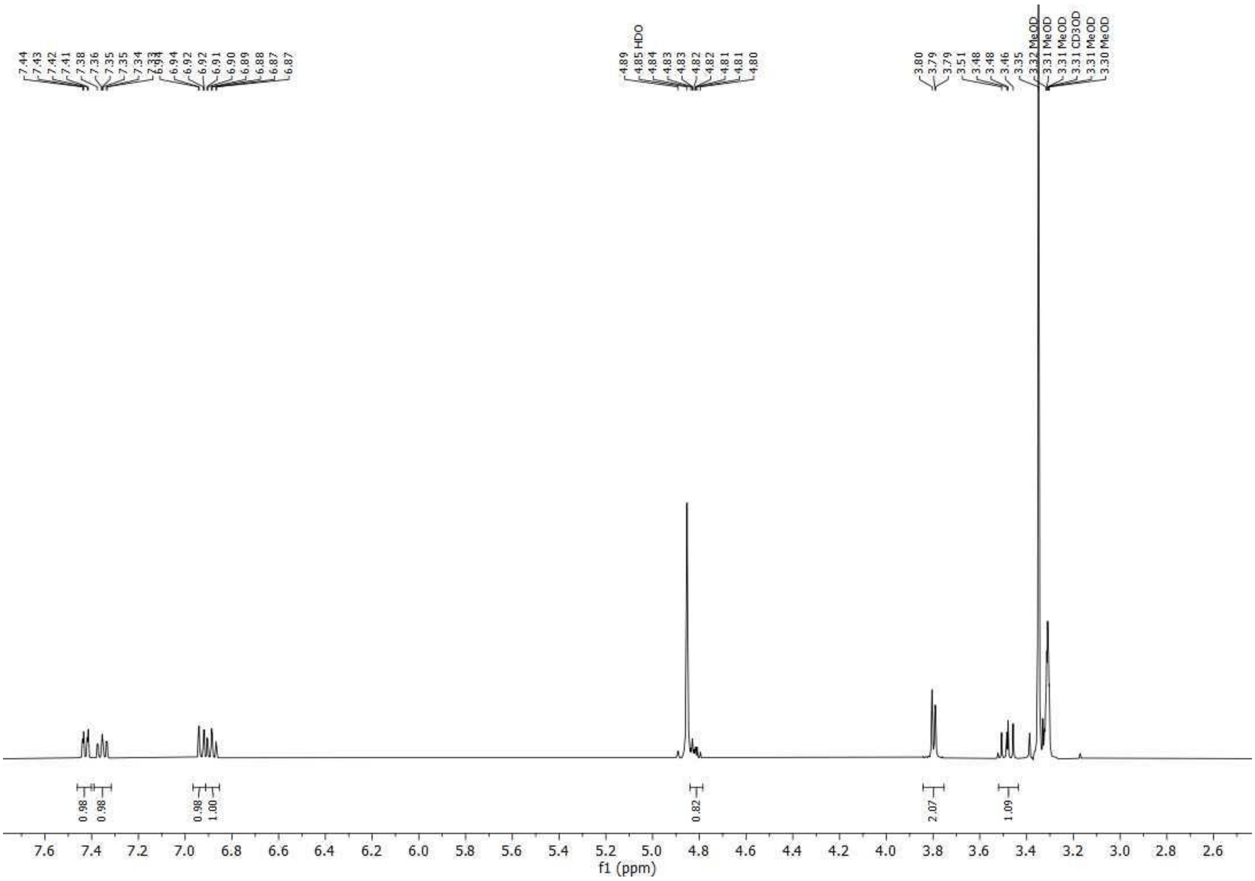

^{1}H NMR in CD_3OD of synthetic (*R*)-aerugine.

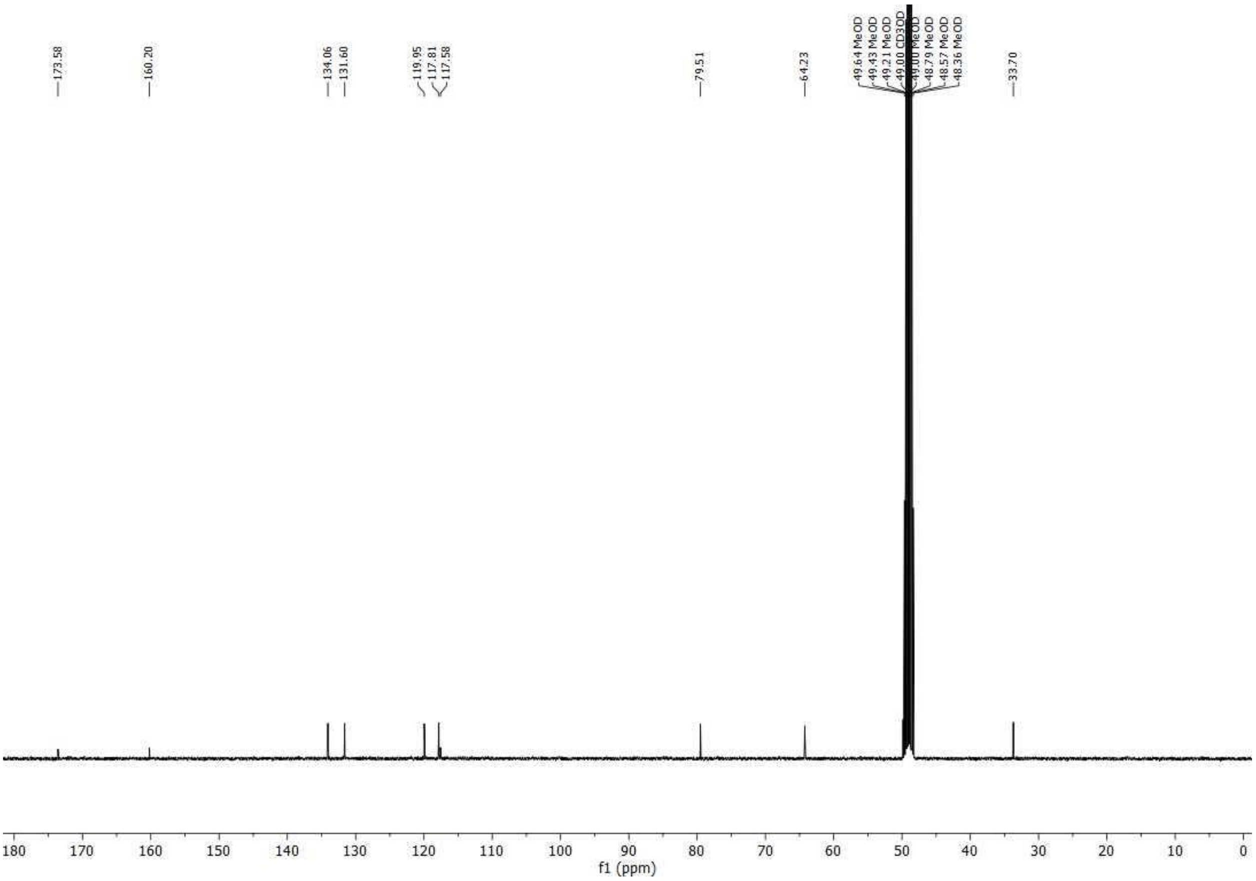

^{13}C NMR in CD_3OD of synthetic (*R*)-aerugine.

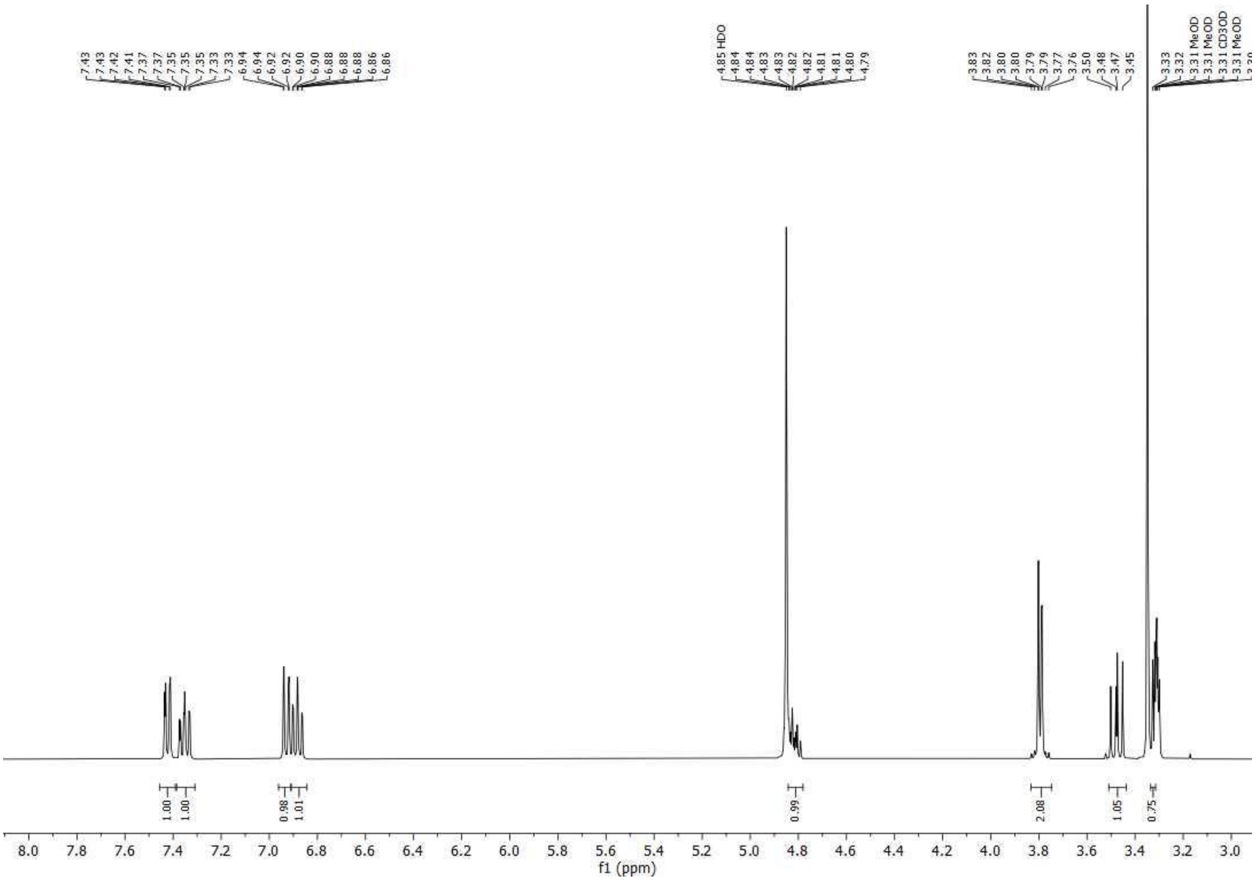

^{1}H NMR in CD_3OD of synthetic (*S*)-aerugine.

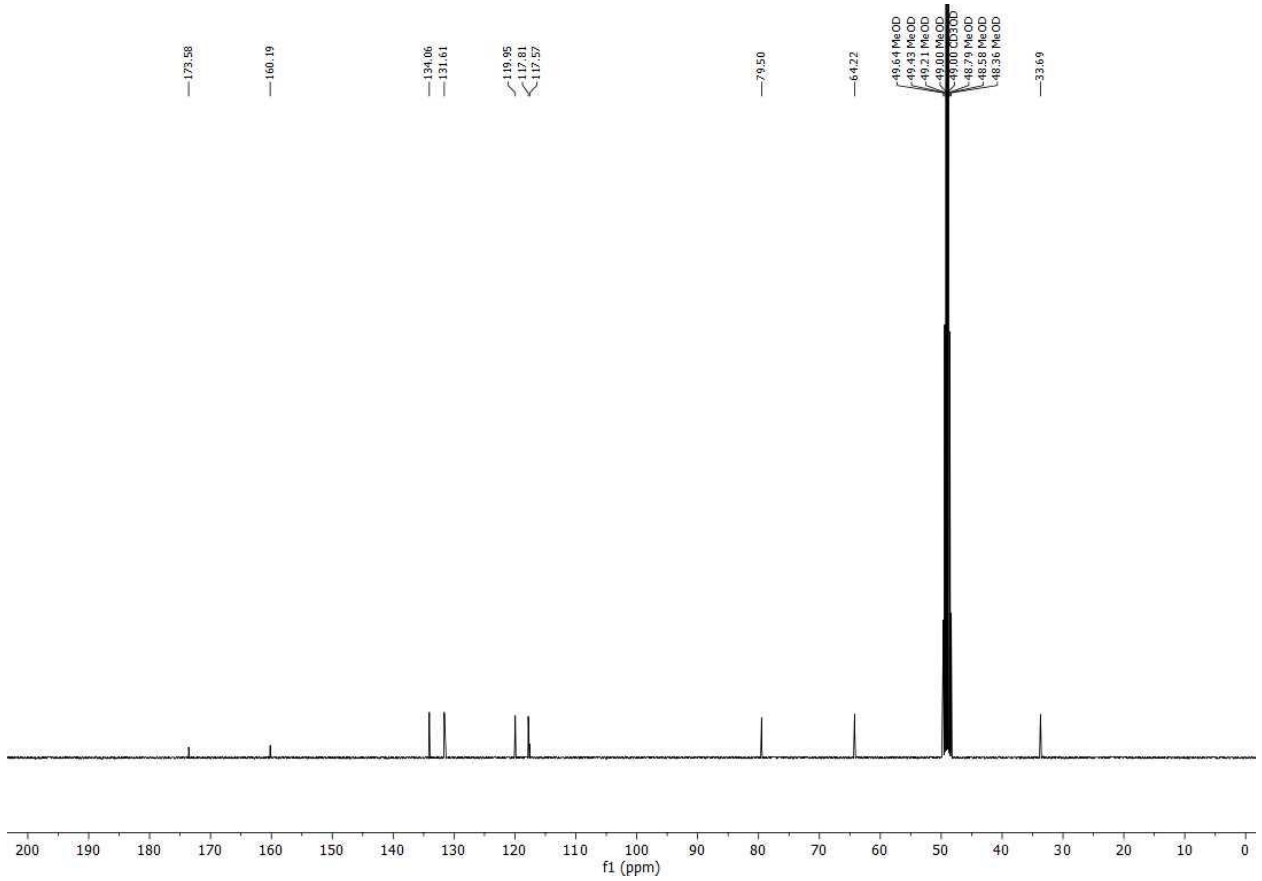

^{13}C NMR in CD_3OD of synthetic (*S*)-aerugine.

Supplementary references

Cooper GM (2000) Transport of Small Molecules, *The Cell: A Molecular Approach*. Sunderland (MA): Sinauer Associates.

de Souza-Fagundes EM, Delp J, Prazeres PDM, Marques LB, Carmo AML, Stroppa PHF, Glanzmann N, Kisitu J, Szamosvàri D, Böttcher T, Leist M, da Silva AD (2018) Correlation of structural features of novel 1,2,3-triazoles with their neurotoxic and tumoricidal properties. *Chem Biol Interact* 291, 253-263.

Delp J, Funke M, Rudolf F, Cediel A, Bennekou SH, van der Stel W, Carta G, Jennings P, Toma C, Gardner I, van de Water B, Forsby A, Leist M (2019) Development of a neurotoxicity assay that is tuned to detect mitochondrial toxicants. *Arch Toxicol* 93, 1585-1608.

Drechsel H, Stephan H, Lotz R, Haag H, Zähner H, Hantke K, Jung G (1995) Structure elucidation of yersiniabactin, a siderophore from highly virulent Yersinia strains. *Liebigs Ann Chem* 1995, 1727-1733.

Endo S, Escher BI, Goss KU (2011) Capacities of membrane lipids to accumulate neutral organic chemicals. *Environ Sci Technol* 45, 5912-5921.

Igarashi Y, Asano D, Sawamura M, In Y, Ishida T, Imoto M (2016) Ulbactins F and G, Polycyclic Thiazoline Derivatives with Tumor Cell Migration Inhibitory Activity from Brevibacillus sp. *Org Lett* 18, 1658-1661.

Inahashi Y, Zhou S, Bibb MJ, Song L, Al-Bassam MM, Bibb MJ, Challis GL (2017) Watasemycin biosynthesis in Streptomyces venezuelae: thiazoline C-methylation by a type B radical-SAM methylase homologue. *Chem Sci* 8, 2823-2831.

Lin Z, Antemano RR, Hughen RW, Tianero MD, Peraud O, Haygood MG, Concepcion GP, Olivera BM, Light A, Schmidt EW (2010) Pulicatins A-E, neuroactive thiazoline metabolites from cone snail-associated bacteria. *J Nat Prod* 73, 1922-1926.

Liu H, Wang L, Lv M, Pei R, Li P, Pei Z, Wang Y, Su W, Xie XQ (2014) AlzPlatform: an Alzheimer's disease domain-specific chemogenomics knowledgebase for polypharmacology and target identification research. *J Chem Inf Model* 54, 1050-1060.

Marques LB, Ottoni FM, Pinto MCX, Ribeiro JM, de Sousa FS, Weinlich R, de Victo NC, Kisitu J, Holzer AK, Leist M, Alves RJ, Souza-Fagundes EM (2020) Lapachol acetylglycosylation enhances its cytotoxic and pro-apoptotic activities in HL60 cells. *Toxicol In Vitro* 65, 104772.

Pearce RG, Setzer RW, Davis JL, Wambaugh JF (2017) Evaluation and calibration of high-throughput predictions of chemical distribution to tissues. *J Pharmacokinet Pharmacodyn* 44, 549-565.

Sasaki O, Igarashi Y, Saito N, Furumai T (2002) Watasemycins A and B, new antibiotics produced by Streptomyces sp. TP-A0597. *J Antibiot (Tokyo)* 55, 249-255.

Seipke RF, Song L, Bicz J, Laskaris P, Yaxley AM, Challis GL, Loria R (2011) The plant pathogen Streptomyces scabies 87-22 has a functional pyochelin biosynthetic pathway that is regulated by TetR- and AfsR-family proteins. *Microbiology (Reading)* 157, 2681-2693.

Shindo K, Takenaka A, Noguchi T, Hayakawa Y, Seto H (1989) Thiazostatin A and thiazostatin B, new antioxidants produced by Streptomyces tolurosus. *J Antibiot (Tokyo)* 42, 1526-1529.

Winiwarter S, Bonham NM, Ax F, Hallberg A, Lennernäs H, Karlén A (1998) Correlation of human jejunal permeability (in vivo) of drugs with experimentally and theoretically derived parameters. A multivariate data analysis approach. *J Med Chem* 41, 4939-4949.

Youard ZA, Mislin GL, Majcherczyk PA, Schalk IJ, Reimmann C (2007) Pseudomonas fluorescens CHA0 produces enantio-pyochelin, the optical antipode of the Pseudomonas aeruginosa siderophore pyochelin. *J Biol Chem* 282, 35546-35553.
